# Unravelling the Role of Oat β‑Glucan on the Surface Behavior of an Oat Protein-Rich Coextract: An Enzymatic Approach

**DOI:** 10.1021/acs.biomac.6c00335

**Published:** 2026-06-11

**Authors:** Jennifer McLauchlan, Hans Bolinsson, Lars Nilsson, Nick Sirijovski, Arwen I. I. Tyler, Caroline Orfila, Anwesha Sarkar

**Affiliations:** † Food Colloids and Bioprocessing Group, School of Food Science and Nutrition, 4468 University of Leeds, Leeds LS2 9JT, United Kingdom; ‡ Department of Process and Life Science Engineering, Faculty of Engineering LTH, 5193 Lund University, Lund 221 00, Sweden; § Global Oatly Science and Innovation Centre, Oatly AB, Science Village, Lund 224 84, Sweden; ∥ National Alternative Protein Innovation Centre (NAPIC), Leeds LS2 9JT, United Kingdom

## Abstract

Despite extensive research linking oat β-glucan (OBG) to improvements in cardiometabolic health, little is known about how OBG influences the structure and surface behavior of oat protein. To this end, enzymatic hydrolysis of an oat protein-β-glucan coextract (OPBG) from defatted oat flour was performed. The effect of OBG cleavage was characterized using asymmetric flow field-flow fractionation with ultraviolet–visible (UV–vis) and in situ fluorescence detection, allowing for deconvolution of protein and OBG signals. Partial OBG cleavage increased oat protein dispersibility and accelerated adsorption to the air–water interface, albeit partly driven by thermal treatment during enzymatic hydrolysis. Although full cleavage resulted in protein aggregation, all OPBG samples offered decreased air–water surface tension and faster solid surface-water adsorption kinetics compared to oat protein isolate (OPI). Novel findings highlight that oat protein surface behavior can be adjusted by omitting isoelectric precipitation, while retaining inherent OBG in the system to ultimately reduce protein–protein hydrophobic aggregation.

## Graphical abstract

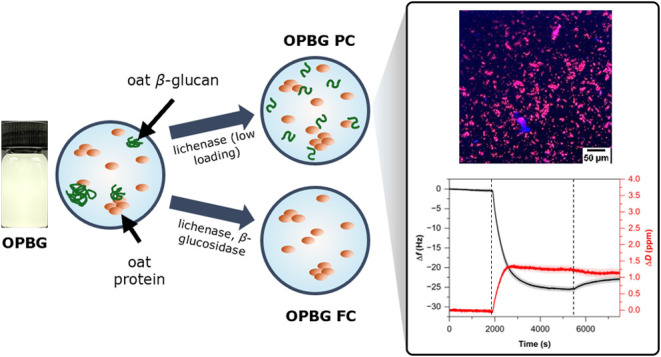

## 1 Introduction

Oats (*Avena sativa*) have been consumed by humans for thousands of years^1^ and are recognized as an affordable crop with low environmental footprint and a relatively high protein content compared to other cereals.^2^ Despite its long history as a cultivated grain, understanding of oat protein is still in its early stages compared to other plant proteins.^3^ A key challenge for wider utilization is that oat proteins suffer from notoriously poor solubility after isolation via isoelectric precipitation (IEP), largely due to protein–protein hydrophobic aggregation at neutral to mildly acidic pH.^4^ This effect is exacerbated in kilned oats, with reports of reduced protein solubility compared to non-kilned equivalents.^22,52^

Although targeting oat kilning is becoming a common approach to reduce protein aggregation and improve extractability,^5,6^ the interaction of oat protein with other naturally present biomacromolecules in the oat matrix and the effect this has on the protein’s aggregation properties, dispersibility and surface behavior remain poorly understood. While solubility refers to the complete molecular dissolution of proteins in an aqueous solvent, dispersibility describes the extent to which insoluble aggregates can be evenly suspended throughout the medium. Therefore, dispersibility is a critical parameter for determining functional performance of plant protein dispersions, where full dissolution is typically not achieved. Understanding how other naturally present components, such as dietary fiber, influence protein structure and surface behavior is also important for the formulation of oat protein products, with likely implications in protein digestibility^7^ and colloidal interactions.

Herein, we focus on how oat protein interacts with oat β-glucan (OBG), the predominant soluble fiber in oats, which consists of linear d-glucopyranosyl residues linked by mixed β-(1→3) and β-(1→4) glycosidic bonds.^8^ Previous work by Zielke et al. demonstrated a direct association between oat protein and OBG with no evidence of covalent bonding, indicating that interactions are primarily governed by ionic forces or hydrogen bonding.^9^ Consistent with this, a molecular modeling study of mixed OBG–soy protein isolate gels revealed that hydrogen bonding was the dominant interaction, with the mixed gels leading to reduced gelation time compared to pure protein gels.^10^ Glycosylation of pea protein isolate with OBG has also been shown to improve emulsification properties,^11^ warranting investigation into whether similar effects occur in oat protein systems.

We previously characterized a protein rich extract from oats which contains OBG as the other major component.^12^ After subjecting the sample to different pH and ionic strength conditions, we suggested a hydrogen bonding-driven interaction between the two constituents. This oat protein-β-glucan coextract (OPBG) differs from oat protein isolates (OPI) traditionally studied in the literature by omission of the IEP and the lyophilization step, the former inducing protein precipitation after lowering the pH to the globulin isoelectric point (pH 4.0–5.0). While useful for concentrating the protein to high levels of purity (>90%), by definition IEP induces protein aggregation in the system and the conditions of IEP have been reported to impact protein solubility.^13^ Meanwhile, lyophilization is routinely used in plant protein studies due to a higher preservation of protein structure compared to other drying methods,^14^ with protein structural changes induced by lyophilization reported as fully reversible upon rehydration for animal derived proteins.^15^ Although the effects of drying oat protein are not well understood and may induce structural changes, we anticipate that the IEP step is the primary factor driving protein–protein aggregation in OPI.^16,17^

OPBG was obtained by performing protein extraction in an alkaline/salt solution, followed by neutralization, dialysis and storage in frozen liquid form. Although OPBG showed a pH dependent morphology and similar protein properties to isolates in the literature,^12^ what remained unclear is whether OBG had a direct impact on the structure and functionality of the extracted proteins. To provide clarity, an in-depth investigation into whether and how naturally occurring fiber affects oat protein properties is long overdue.

One of the challenges with understanding this complex sample is deconvoluting the protein and OBG data, both of which contain aggregates ranging from nanometres to tens of microns.^12^ A commonly used separation technique to analyze complex samples is asymmetric flow field-flow fractionation (AF4) due to its compatibility with various materials, from nanoparticles^18^ to biomacromolecules.^19,20^ AF4 provides separation of components without the use of a stationary phase, thus offering a gentler separation which reduces the effective shear experienced by sample aggregates. The technique can be coupled to various detectors including UV–vis, multiangle light scattering (MALS) and fluorescence light detection (FLD) to provide comprehensive information on size and composition. Notably, AF4 has previously been used to analyze complex protein samples including wheat gluten proteins,^21^ oat protein extracts^5,22^ and extracts from barley malts and brewer’s spent grain.^23^ The latter study similarly utilized a combination of UV–vis absorption for protein identification and in-line staining with fluorescence detection for OBG.^24,25^ Calcofluor White is typically employed as the stain for OBG detection in separation techniques,^24,25^ as it binds to polysaccharides containing β-(1→3) and β-(1→4) linkages.^26^

Herein, we employ systematic enzyme treatment with lichenase and β-glucosidase to obtain partially and fully cleaved OBG in an oat protein-rich extract (OPBG), with the aim of (1) understanding the influence of dietary fiber OBG on the protein structure and aggregation state, and (2) determining whether there is a functional benefit for surface behavior in using OPBG over the traditional OPI. Surface behavior was studied here at the air–water interface, using the pendant drop method, and the hydrophobic solid surface-water interface, using quartz crystal microbalance with dissipation monitoring (QCM-D). While enzyme treatment has previously been used to monitor changes in pea protein structure upon cleavage of fiber,^27^ to our knowledge this is the first time this approach has been implemented on an oat protein extract.

## 2 Experimental Methods

### 2.1 Materials

Whole oat groats, kindly provided by Oatly AB (Lund, Sweden), were derived from a commercial blend of European oats harvested in 2022 and dehulled and kilned by a mill in Belgium. Soxhlet extraction was performed using Whatman grade 603 Standard Cellulose Extraction Thimbles with dimensions 33 × 80 mm^2^ and 1.5 mm thickness (Scientific Laboratory Supplies, Nottingham, U.K.) and hexane (ReagentPlus, ≥ 99%, Sigma-Aldrich, Gillingham, U.K.). Tris base (Thermo Fisher Scientific, Loughborough, U.K.), HEPES (Sigma-Aldrich, Gillingham, U.K.) and dialysis tubing with a 10 000 g/mol cutoff (Scientific Laboratory Supplies, Nottingham, U.K.) were used during the extraction. Water with a resistivity of 18.2 M Ω·cm at 25 °C was retrieved from a Milli-Q apparatus for all experiments in this study (Millipore Corp., Bedford, MA).

The enzymes used for hydrolysis of OBG were lichenase from *Bacillus subtillis* and β-glucosidase from *Agrobacterium* sp. with reference codes of E-LICHN and E-BGOSAG respectively (Megazyme, Bray, Ireland). Stock enzyme solutions were prepared with 10 mmol/L HEPES buffer (pH 7.0), which will subsequently be referred to as HEPES buffer. The solutions were aliquoted and stored at −20 °C until required.

Fast Green (dye content ≥ 85%, Sigma-Aldrich, Gillingham, U.K.) and Calcofluor White Stain (Sigma-Aldrich, Gillingham, U.K.) were used for confocal laser scanning microscopy (CLSM) imaging, the latter containing a mixture of 1 mg/mL Calcofluor White M2R and 0.5 mg/mL Evans blue counterstain. Unless otherwise specified, the chemicals used were of analytical grade.

### 2.2 Methods

#### 2.2.1 Preparation of OPBG and OPI

The preparation of OPBG was based on a previous study^12^ with minor modifications, as shown in Figure 1a. Briefly, oat groats were milled using a coffee grinder (Model F2034238, Krups, Datchet, U.K.) for approximately 1 min and screened through a 500 μm mesh. The resulting oat flour was then defatted via Soxhlet extraction (SOXTHERM, Gerhardt GmbH & Co., Königswinter, Germany) with hexane and allowed to dry in a fume cupboard overnight. The defatted oat flour was dispersed at 1:10 w/v in a solution of 50 mmol/L Tris-HCl buffer and 1.0 mol/L NaCl at pH 8.5. The solution was stirred for 2 h (500 rpm, rt) followed by centrifugation to remove insoluble components (20 °C, 4000×*g*, 20 min). The resulting supernatant will be referred to as oat protein (OP) precursor (Figure 1ai).

**Figure 1:**
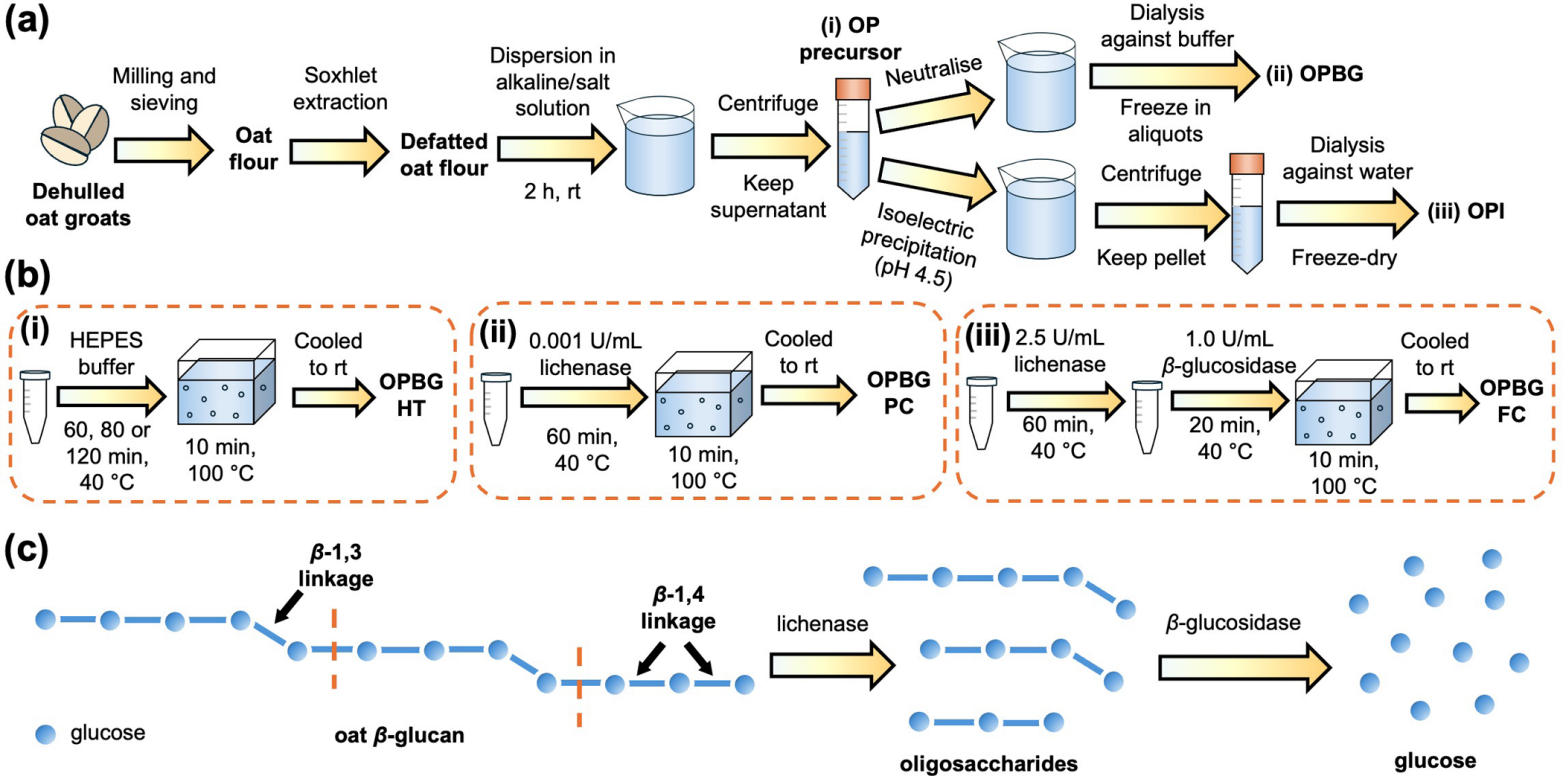
Schematic of oat protein sample preparation and enzymatic hydrolysis of oat β-glucan (OBG) . (a) Summarized preparation protocol used to obtain (i) oat protein (OP) precursor, (ii) oat protein-β-glucan coextract (OPBG), and (iii) oat protein isolate (OPI). (b) Reaction conditions selected to prepare (i) OPBG heat treated (HT) control samples, (ii) OPBG partially cleaved (PC) and (iii) OPBG fully cleaved (FC). All reactions were conducted in HEPES buffer. (c) Schematic showing the hydrolytic action of lichenase and β-glucosidase enzymes on OBG. Approximately 92% of the oligosaccharides resulting from pure OBG digestion with lichenase were found to be made up of trisaccharides and tetrasaccharides.^28^

To obtain OPBG, the OP precursor was neutralized with 1.0 mol/L HCl and dialyzed against HEPES buffer overnight. Single use OPBG aliquots were frozen and stored at −20 °C until required. To obtain OPI, the pH of OP precursor was reduced to pH 4.5 with 1.0 mol/L HCl to induce IEP. Following a second centrifugation step (20 °C, 4000×*g*, 20 min), the pellet was collected, dispersed in water and dialyzed overnight against water to remove any remaining salt. The resulting dialysate was freeze-dried and stored in a desiccator at room temperature until required. Despite multiple batches of OPBG being used for this study, all samples were extracted from the same oat groat starting material. It should be noted that OPBG was dialyzed against HEPES buffer to ensure compatibility with subsequent enzyme reactions. In contrast, OPI was dialyzed against water prior to freeze-drying and later dispersed in 10 mmol/L HEPES buffer, thereby avoiding unintended changes in salt composition.

#### 2.2.2 Enzymatic Cleavage of OBG in OPBG

Figure 1b summarizes the conditions used to prepare fully cleaved (FC) and partially cleaved (PC) OPBG, along with heat-treated (HT) control samples. The enzyme concentrations of lichenase and β-glucosidase for OPBG FC were selected based on the Megazyme β-glucan assay kit (K-BGLU) under AOAC Method 995.16. Details of the hydrolytic action of each enzyme used is drawn schematically in Figure 1c. For OPBG PC, two enzyme concentrations (0.1 and 0.001 U/mL) were tested to determine optimal hydrolysis conditions for this sample (Supporting Information, Figure S1). These enzyme concentrations were selected based on previous work by Roubroeks et al., who conducted partial hydrolysis of purified OBG samples using lichenase.^29^ All reactions were conducted at pH 7.0 and retained at 40 °C using a shaking incubator at 500 rpm (Heidolph Titramax 1000). To stop the reaction, vials were placed in a boiling water bath for 10 min prior to cooling to room temperature. Samples were then stored at 4 °C for up to 48 h prior to analysis.

#### 2.2.3 Asymmetric Flow Field-Flow Fractionation (AF4)

For AF4 experiments, a short channel equipped with a 350 μm spacer and a poly­(ether sulfone) (PES) membrane with a molecular weight cutoff of 10 000 g/mol was used (Wyatt Technology, Dernbach, Germany). An Agilent isocratic pump with an in-line vacuum degasser delivered the carrier liquid to the system and an autosampler was used for sample injection. The carrier liquid was filtered prior to entrance to the channel with a 0.1 μm polyvinylidene fluoride (PVF) membrane for removal of particles and dust. For the OP precursor, the carrier liquid used was 50 mmol/L Tris-HCl (pH 8.5) with 1 mol/L NaCl. For the remaining samples, HEPES buffer with 0.02% NaN_3_ was used as the carrier liquid. All samples were centrifuged at 13,000 rpm (11,337×*g*) at room temperature for 30 min prior to injection.

##### 2.2.3.1 UV–vis and MALS Set Up

An Eclipse 3+ Separation System (Wyatt Technology, Dernbach, Germany) was connected to an Agilent 1100 series UV–vis detector set to 280 nm, followed by a Dawn Helios II MALS detector at 658 nm (Wyatt Technology, Dernbach, Germany). Consistent with a previous oat protein AF4 study,^22^ the molar extinction coefficient for UV absorbance was assumed to be that of bovine serum albumin in aqueous solution (660 mL g^–1^ cm^–1^). Figure 2a shows the programmed detector and cross-flow rates with a sample injection volume of 50 μL. This corresponded to a mass of ∼250 μg for the OP precursor and ∼35 μg for OPBG. Briefly, the focusing step followed a constant cross-flow of 3.5 mL/min and lasted for 12 min. The elution step remained at a fixed cross-flow rate of 3.5 mL/min until 28 min, at which point the cross-flow decreased linearly from 3.5 mL/min and reached 0.1 mL/min at 42 min. The cross-flow was maintained at 0.1 mL/min until 77 min, at which point the cross-flow was set to 0 mL/min. The detector flow rate remained constant at 1 mL/min throughout the experiment. At the end of each experiment, the channel was rinsed without any crossflow for 5 min prior to injection of the next sample.

**Figure 2:**
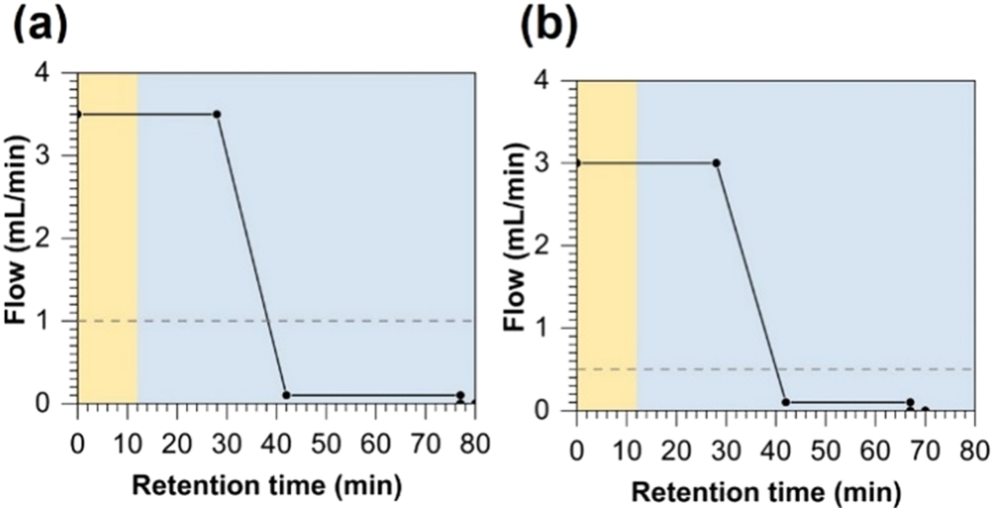
AF4 detector and cross-flow set up used in (a) UV/MALS set up and (b) FLD set up. The yellow shaded area represents the AF4 focusing stage, while the elution step is shaded blue. The set cross-flow is drawn as a solid line, and the dashed line represents the detector flow which remained constant throughout the run. After separation in the AF4 channel in (b), the sample was mixed at a 1:1 ratio with Calcofluor White solution (0.5 mL/min), resulting in an effective detector flow of 1 mL/min prior to reaching the fluorescence detector.

Fitting of the MALS data was performed with the Berry method^30^ using the Astra Software (Wyatt Technology, Dernbach, Germany) to obtain values of molar mass (MM) and radius of gyration (*R*_g_). The Berry model was selected as it gives the most accurate results for nonspherical particles with an *R*_g_ above 50 nm.^31^ Since the samples contained a mixture of two macromolecules eluting at different time points, the MM analysis was conducted twice using refractive index increments (d*n*/d*c*) of bovine serum albumin^32^ (0.186 mL/g) and of pure OBG^33^ (0.146 mL/g) respectively.

##### 2.2.3.2 Fluorescence Light Detection (FLD) Set Up

A separate setup was used for in-line Calcofluor White staining and detection of OBG.^24^ An equal sample volume was injected (50 μL), resulting in an injected mass of ∼35 μg for OPBG samples and ∼250 μg for OP precursor. However, the cross-flow and detector flow rates used for this set up were adapted (Figure 2b). The focusing step had a set cross-flow of 3.0 mL/min and lasted for 12 min. The elution step remained at a fixed cross-flow of 3.5 mL/min until 28 min, at which point the cross-flow decreased linearly from 3.5 mL/min and reached 0.1 mL/min at 42 min. The cross-flow was maintained at 0.1 mL/min until 67 min, at which point it was set to 0 mL/min. The detector flow rate remained constant at 0.5 mL/min throughout.

A solution of 25 mg/mL fluorescent brightener 28 (Sigma-Aldrich, Gillingham, U.K.) in 50 mmol/L Tris-HCl buffer at pH 8.5 was pumped at a flow rate of 0.5 mL/min into a T-junction connected to the outlet of the MALS detector. This resulted in a 1:1 mixture of sample: dye solution upon reaching the fluorescence detector. The excitation and emission wavelengths were set to 415 and 445 nm respectively, and the photomultiplier tube (PMT) fluorescence gain was set to 1.

To measure intrinsic protein Trp fluorescence, the same FLD setup was used but the fluorescence dye solution was replaced with the carrier liquid. The excitation and emission wavelengths were altered to 280 and 340 nm respectively, and the PMT fluorescence gain was increased to 10 to account for the weaker intensity associated with intrinsic protein fluorescence. All fluorescence spectra were then normalized by the subtraction of a blank AF4 run in which 50 μL of carrier liquid was injected into the channel.

Retention times for the AF4 method using FLD (Figure 2b) were transformed for comparison with the UV-MALS method (Figure 2a). This was achieved using AF4 theory^34^ by linear interpolation (OriginPro 2026) from the relationship between analyte diffusion coefficient and retention time. The resulting transformed graphs can be found in Figure S2 of the Supporting Information.

#### 2.2.4 Confocal Laser Scanning Microscopy (CLSM)

The particle morphology was elucidated using an LSM 880 upright confocal microscope and analyzed with Zen Black software (Carl Zeiss MicroImaging, Jena, Germany). Most scattered light was filtered out by setting the pinhole diameter to 1 Airy Unit (AU). Fast Green (1 mg/mL in water) for protein staining and Calcofluor White (1 mg/mL) for OBG staining were added to the sample, then allowed to equilibrate in the dark for a minimum of 15 min prior to measurement. Excitation of dyes was conducted at 380 nm for Calcofluor White (blue) and 633 nm for Fast Green (red). All images were subsequently analyzed with Fiji ImageJ^35^ software.

#### 2.2.5 Apparent Viscosity Flow Curves

Apparent viscosity (η) was measured using an MCR-302 stress-controlled rheometer (Anton Paar, Hertfordshire, U.K.). The rheometer was equipped with an Anton Paar cylindrical double gap cylinder geometry (DG27/T200/SS) for measuring low viscosity samples. The geometry contained a bob outer diameter of 27.001 mm, a bob inner diameter of 24.997 mm, a cup outer diameter of 29.282 mm and a cup inner diameter of 23.039 mm. All measurements were conducted at 25 °C and the sample η was measured as the shear rate (γ̇) increased from 0.5 to 100 s^–1^ with a gap size of ∼1 mm. The apparent viscosity flow curves of three independent samples (*n* = 3) were measured and plotted as a mean.

#### 2.2.6 Air–Water Interfacial Tension

The surface tension at the air–water interface was measured using an OCA 24 drop shape tensiometer (Dataphysics Instruments, Filderstadt, Germany). A 20 μL drop of sample without further dilution (protein concentration of 4.4 mg/mL) was suspended at the tip of a syringe (DS500 GT, 1.65 nm) in air at room temperature (25 °C) and monitored for 6000 s. The interfacial tension over time was calculated using the Young–Laplace equation with droplet shape fitting using the dpiMAX software (Dataphysics Instruments, Filderstadt, Germany). All measurements were conducted in triplicate (*n* = 3) using a minimum of two independently prepared samples.

#### 2.2.7 Adsorption Kinetics at the Air–Water Interface

The kinetics of protein adsorption can be described in three stages:^36^ (i) diffusion controlled movement of proteins from bulk to interface (*k*_1_), (ii) penetration of proteins at the interface (*k*_2_) and (iii) conformational reorganization of proteins at the interface (*k*_3_). Information on the rate constants for each stage was obtained from the dynamic surface tension curves by first converting to interfacial pressure (π) with eq 1, where γ_0_ is the interfacial tension of the HEPES buffer in air and γ is the interfacial tension of the oat protein sample.

1 $$\pi \;\left(\mathrm{mN}/m\right)=\gamma_{0}-\gamma$$

The initial slope of a plot of π against *t* ^1/2^ was used to determine the value of *k*_1_, following the modified Ward & Tordai^37,38^ in eq 2, where *C*_0_ is the protein concentration, *k*_B_ is the Boltzmann constant, *T* is the absolute temperature and *D* is the diffusion coefficient.

2 $$\pi =2C_{0}k_{B}T\;{\left(\mathrm{Dt}/3.14\right)}^{1/2}$$

The penetration (*k*_2_) and rearrangement (*k*_3_) first order rate constants were determined by eq 3.^36^ The slope of the first linear region was taken as the value of *k*_2_ and the slope of the second linear region was reported as *k*_3_.

3 $$\ln\left(\pi_{f}-\pi_{t}\right)/\left(\pi_{f}-\pi_{0}\right)=-k_{i}t$$

#### 2.2.8 Adsorption onto Hydrophobic Solid Surfaces

##### 2.2.8.1 Preparation and Cleaning of Polydimethylsiloxane (PDMS) Coated Sensors

The preparation of quartz crystal microbalance with dissipation monitoring (QCM-D) sensors was based on previous work.^39,40^ Briefly, Q-Sense SiO_2_ sensors (QSX-303, Biolin Scientific, Sweden) were cleaned using UV/ozone treatment for 15 min and immersed in sulfuric acid (95%) for 1 h. After sonicating twice in water for 10 min, these were fully dried under a nitrogen stream. Sensors were then immersed in RCA solution (5:1:1 v/v/v water: ammonia: hydrogen peroxide) at 80 °C for 10 min, followed by 3 × 10 min sonication cycles in water. The clean sensors were dried under a nitrogen stream then spin-coated with 150 μL of 0.5 wt % polydimethylsiloxane (PDMS) in toluene at 5,000 rpm for 30 s and an acceleration of 2500 rpm/s. Coated sensors were left to cure overnight in a vacuum oven at 80 °C. Prior to measurements, sensors were immersed in toluene (30 s), isopropyl alcohol (30 s) and water (5 min). Solvents were then left to evaporate for 1 h in the fume cupboard.

##### 2.2.8.2 Experimental Set Up

The adsorption behavior of oat protein samples was measured using QCM-D (Q-Sense, Biolin Scientific, Sweden). Samples entered the chamber held at 25 °C using a peristaltic pump (Ismatec, Germany) at a set flow rate of 100 μL min^–1^. HEPES buffer was degassed using an ultrasonic bath (XUB18, Grant Instruments, Cambridge, U.K.) for 10 min to remove bubbles, then flowed through the system for 30 min to achieve a stable baseline. Next, samples dissolved at 0.1 mg/mL protein were allowed to adsorb until an equilibrium state was reached (Δ*f* not changing more than 1–2 Hz for 30 min). A final HEPES buffer rinse was used to remove weakly adsorbed sample. The resulting changes in frequency (Δ*f*) and dissipation (Δ*D*) over time were collected by the QSoft software (Q-Sense, Biolin Scientific, Sweden) and analyzed with Dfind (Q-Sense, Biolin Scientific, Sweden).

#### 2.2.9 Statistics

Unless otherwise specified, reported values represent the mean ± standard deviation of a minimum of three data sets (*n* = 3), with at least one data set derived from an independently prepared sample. All data fitting and analysis was performed using OriginPro 2025. Significant differences between independent samples were assessed using an unpaired Student’s *t*-test. A *p* value of less than 0.05 was considered significant and marked with an asterisk (*). Values of *p* < 0.01 and *p* < 0.001 were marked with two (**) and three (***) asterisks, respectively.

## 3 Results and Discussion

### 3.1 AF4 Characterization of OPBG and OP Precursor

AF4 was employed to characterize the nanoscale structure of OPBG prior to enzyme treatment. For details on the proximate composition and recovery values of protein and OBG in OPBG, we refer the reader to our previous study.^12^ Please note that retention times are not strictly identical when comparing data obtained from the UV/MALS and FLD set up due to differences in Figure 2. However, a transformation of the FLD retention time using AF4 theory was attempted to increase comparability to the UV/MALS set up (Supporting Information, Figure S2) and minimal change was observed in the composition of the AF4 populations discussed herein.

Resulting from a marked decrease in the solubility of oat protein after neutralization and dialysis in a low salt buffer,^4,12^ approximately 95% of the total protein content in OPBG was lost upon centrifugation prior to injection into the instrument (Supporting Information, Figure S3). Consequently, an analysis of the OP precursor (Figure 1 ai) was conducted to understand the composition and structure of the sample following protein dissolution in the alkaline/salt extraction buffer. The loss of solubilized protein in OPBG compared to the OP precursor was confirmed by a weakened intensity in the light scattering and UV signals (Figure 3a). Inset photographs in Figure 3b showcase the change in sample appearance from pale yellow to turbid white after the transition from OP precursor to OPBG. This change in turbidity once again displays the dramatic decrease in protein solubility upon neutralization and removal of salt. The color of OP precursor also suggests the presence of other components, likely phenolic compounds in oats (e.g., avenanthramides), which are removed during dialysis and can cause yellowing at alkaline pH.^41^

**Figure 3:**
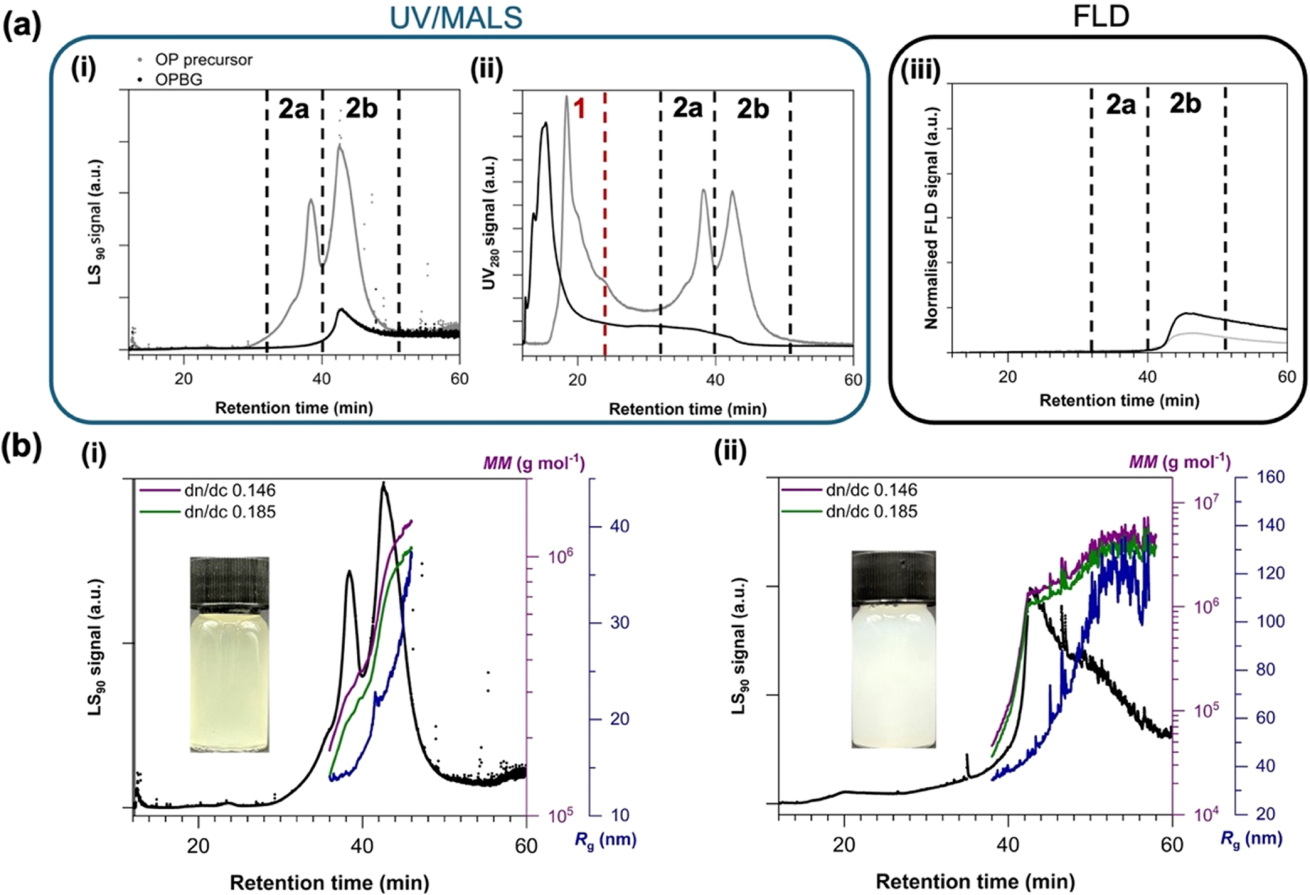
AF4 fractograms of OPBG and OP precursor. (a) AF4 fractograms of (i) the light scattering obtained at a detector angle of 90° (LS_90_), (ii) UV signal at 280 nm and (iii) fluorescence light detection obtained using the *in-line* Calcofluor White staining fluorescence (FLD) set up. The data set was truncated at 60 min, since only this portion of the signal was used for the analysis. (b) Molar mass (MM) and radius of gyration (*R*_g_) values obtained from the light scattering data analysis for (i) OP precursor and (ii) OPBG. Two d*n*/d*c* values were used for MM analysis. Inset: sample photographs captured of (i) OP precursor and (ii) OPBG prior to centrifugation.

Two regions of interest in Figure 3a were assigned based on existing oat protein literature, i.e., smaller monomeric proteins (peak 1), globulins and soluble aggregates (peaks 2a and 2b).^22^ Please note that large MM components were also observed post-elution but could not be analyzed. No light scattering signal was observed for peak 1 which suggests there are small (<15 nm), soluble components eluting that strongly absorb UV light. Since both the dialysis membrane and the AF4 membrane had a molecular weight cutoff of 10,000 g/mol, it is unlikely that solubilized polyphenols were retained in the channel during the measurement. Therefore, peak 1 was assigned to either small monomeric proteins or protein-polyphenol complexes which are below the MALS limit of detection.

In peak 2a, the radius of gyration (*R*_g_) obtained was ∼14 nm with a molar mass (MM) of ∼300,000 g/mol after analysis using the protein d*n*/d*c* (0.186 mL/g). Based on previous light scattering and MM analysis of oat protein systems,^22,32^ we predict that solubilized hexameric oat 12S globulin, the major protein fraction in oats, is eluting here. At this retention time, no Calcofluor White FLD signal was observed, indicating that this peak is mostly composed of protein.

Light scattering data from peak 2b gave an *R*_g_ of 22 nm at the peak maximum, with values increasing to ∼ 38 nm and a MM of 600,000–800,000 g/mol. Both UV and Calcofluor White FLD signals were detected at this retention time, which suggests that this peak consists of a coelution of protein aggregates and OBG. The presence of oat globulins and their aggregates in peaks 2a and 2b was further confirmed by Trp FLD measurements of the OP precursor (Supporting Information, Figure S4), which minimizes light scattering artifacts associated with UV absorbance at 280 nm.^42^ Peaks 2a and 2b showed overwhelming fluorescence intensities compared to peak 1, consistent with globulins comprising approximately 70–80% of the total protein content in oats.^43^

Despite our previous study reporting partial colocalization of protein and OBG signal in OPBG^12^ and the reported ability of OBG to associate with various proteins,^9,44^ Figure 3 alone does not confirm whether there is a direct association between both components or simply a coelution. We therefore employed enzymatic cleavage of OBG in OPBG as method of confirming this hypothesized interaction. If OBG and protein merely coexist in the extract, enzymatic cleavage of OBG would not alter the protein elution profile. Conversely, a shift in the protein elution behavior following OBG cleavage would indicate an association between the two components.

### 3.2 Effect of Heat and Enzymatic Treatment on OPBG Structure

Control samples without enzyme present were first tested in Figure 4 to understand how the incubation conditions, which were necessary for a successful enzymatic reaction, affect the structural properties of OPBG. For more details on the heating conditions used, see Figure 1b. Only the 60 min and 120 min incubation time points were included in Figure 4, as these represent the shortest and longest incubation times used when establishing the optimal enzyme conditions.

**Figure 4:**
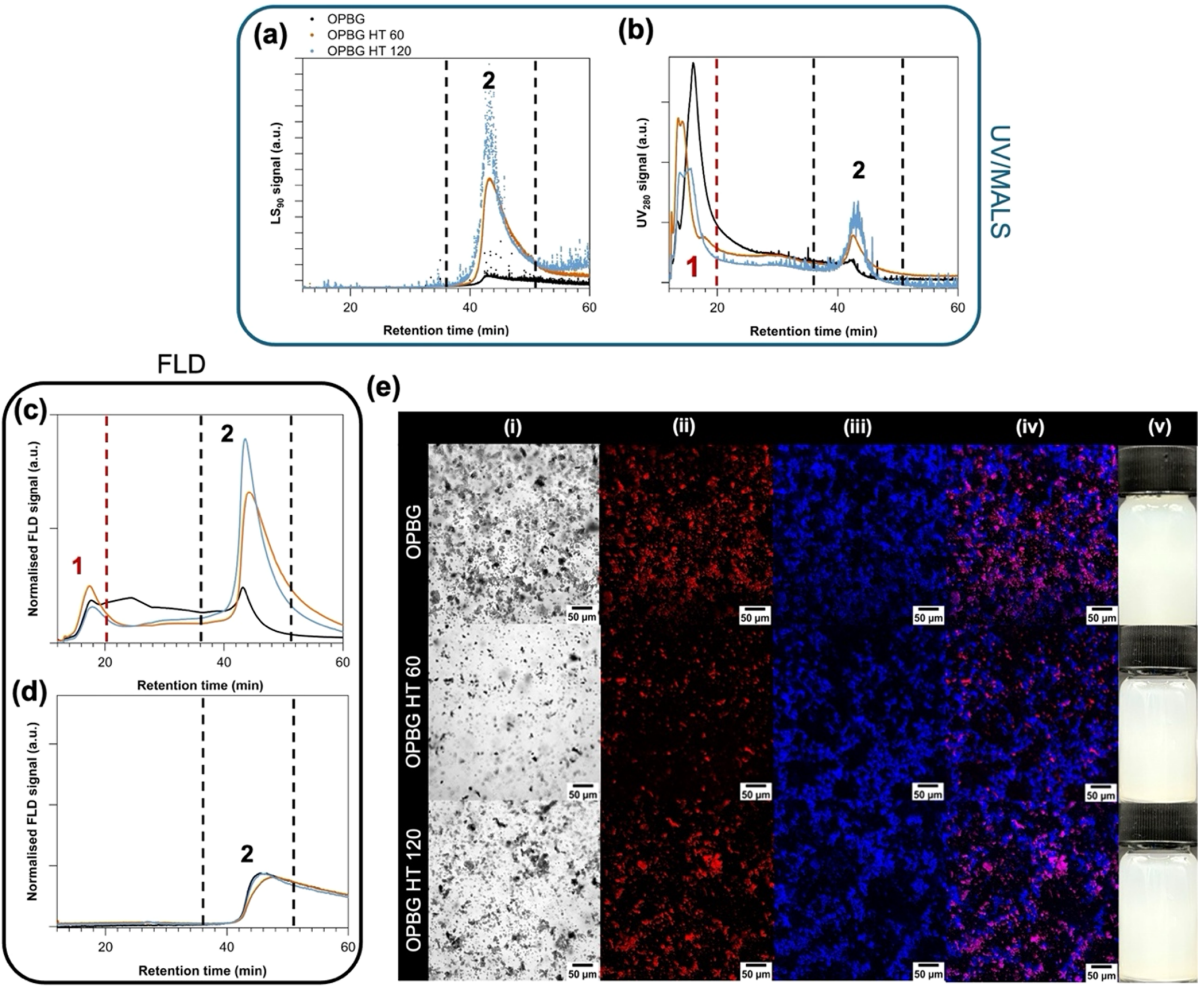
Effect of heat treatment (HT) on the structure of OPBG without the presence of enzyme. AF4 fractograms of HT control samples showing (a) light scattering obtained at 90° (LS_90_), (b) UV–vis signal captured at 280 nm, (c) Calcofluor White fluorescence using the FLD set up, and (d) intrinsic protein tryptophan (Trp) fluorescence captured using the FLD set up. The data set was truncated at 60 min, as only this portion of the signal was used for analysis. (e) Confocal laser scanning micrographs showing the effect of heat treatment on particle morphology. Brightfield images are shown in (i). Samples were stained with Fast Green and Calcofluor White using excitation wavelengths of (ii) 633 nm and (iii) 380 nm, respectively. Fluorescence channels (ii) and (iii) were merged to create (iv). The macroscale appearance of samples was captured directly after mixing in (v).

As a function of incubation time, we observed a decrease in the UV signal in peak 1 combined with an increase in both the UV and light scattering signal intensity in peak 2 (Figure 4a,b). Since the Calcofluor White FLD profile remained constant for all HT samples (Figure 4c), these changes were attributed to the proteinaceous components of OPBG. Trp FLD measurements (Figure 4d) verified the amplified protein signal in peak 2, but only showed small differences in the intensity of peak 1. This suggests that the apparent changes in UV intensity in peak 1 may be a product of scattering artifacts. When combined with the CLSM micrographs (Figure 4e), which showed a reduction in protein aggregates upon heat treatment, the AF4 results suggest that heat treatment helps to improve the dispersibility of proteins which were previously trapped in large aggregates.

It is well documented that extreme heat treatment of protein solutions results in protein aggregation and reduced solubility resulting from irreversible protein denaturation and unfolding of hydrophobic groups.^45^ Conversely, mild heating at temperatures which do not cause irreversible protein denaturation can often lead to increased dispersibility.^46,47^ This is caused by an increase in the kinetic energy of the protein molecules which enhances their ability to interact with the solvent, effectively opening up the structure. Previous work has reported a high thermal denaturation (*T*_d_) temperature for oat globulin (110 °C) and oat albumins (87 °C),^48^ both of which are above the sample incubation temperature (40 °C) used in this study. Therefore, we hypothesize that the dispersibility of oat protein increases under the mild heating conditions applied, releasing some protein from larger particulate aggregates.

However, a more severe heating step (100 °C, 10 min) was used to inactivate the enzymes after the incubation period. To confirm whether protein denaturation occurred, circular dichroism spectra (Supporting Information, Figure S5) were collected before and after heat treatment. Secondary structure data showed minimal change after heat treatment, providing no signs of irreversible unfolding under the conditions used for this study. Similarly, no difference was observed after conducting sodium dodecyl sulfate polyacrylamide gel electrophoresis (SDS-PAGE) on samples before and after heat treatment (Supporting Information, Figure S6).

The effect of OBG hydrolysis on OPBG structure was plotted in Figure 5. Characterization of OPBG PC revealed a shift in the Calcofluor White FLD signal (Figure 5c) into a defined peak, with a smaller retention time compared to the HT controls (Figure 4c) and a loss of postelution fluorescence signal (Supporting Information, Figure S2). We attribute this to successful partial enzymatic hydrolysis of the fiber, causing a reduction in OBG molecular weight to approximately 7000 g/mol when using d*n*/d*c* 0.146 (Supporting Information, Figure S7, Table S8). The direct effect of lichenase treatment on OBG size was confirmed by the narrowing and shifting of peak 2 toward shorter retention times as a function of enzyme incubation time (Supporting Information, Figure S1).

**Figure 5:**
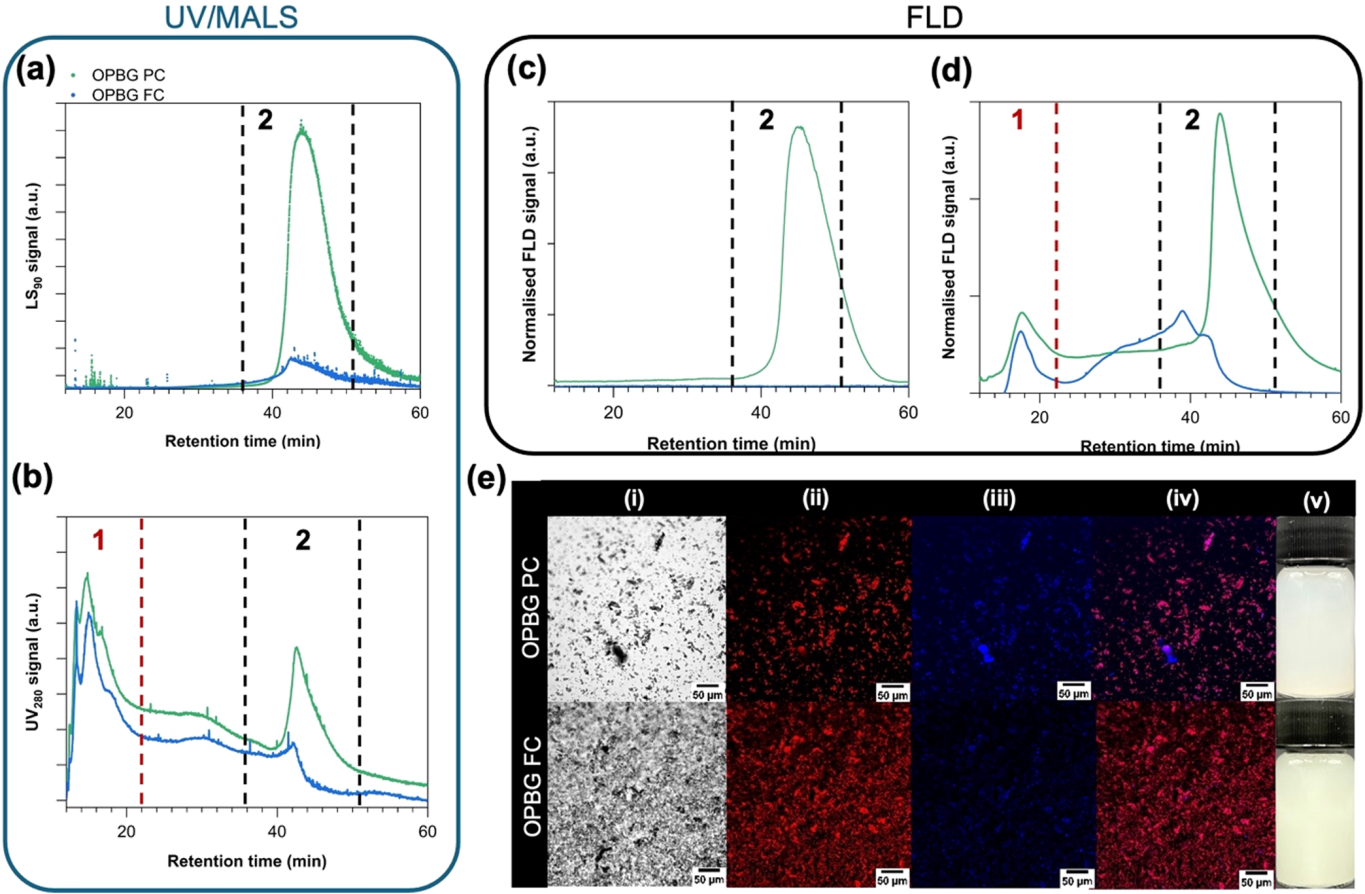
Effect of enzymatic cleavage of OBG on OPBG structure. AF4 fractograms of enzyme treated samples showing (a) light scattering profile obtained at 90° and (b) UV signal captured at 280 nm. The (c) Calcofluor White fluorescence and (d) intrinsic protein Trp fluorescence profiles were captured using the FLD set up. The data set was truncated at 60 min, as only this portion of the signal was used for analysis. (e) Confocal laser scanning micrographs of partially cleaved (PC) and fully cleaved (FC) samples. Brightfield images are shown in (i). Samples were stained with Fast Green and Calcofluor White using excitation wavelengths of (ii) 633 nm and (iii) 380 nm, respectively. Fluorescence channels (ii) and (iii) were merged to create (iv). The macroscale appearance of samples captured directly after mixing is shown in (v).

A signal increase in peak 2 was also observed for OPBG PC in the UV (Figure 5b) and Trp FLD (Figure 5d) fractograms, which surpassed the peak intensities of HT 60 in Figure 4. After integrating the Trp FLD signals (Supporting Information, Figure S9), we observed an increase of 36% in OPBG PC relative to OPBG, indicating improved protein dispersibility. The changes to the protein signal upon OBG hydrolysis confirm that the fiber and the protein are intrinsically associated, with partial cleavage likely increasing dispersibility by releasing protein which was previously trapped in large protein/fiber associated networks. Nevertheless, heat treatment was an important driver for this release which should not be underestimated (Figure 4).

As predicted, no signal was obtained in the Calcofluor White FLD spectrum for OPBG FC (Figure 5c) which confirmed that all the OBG was cleaved to below the limit of Calcofluor White detection (approximately 40 000 g/mol for low salt buffer^49^). For both OPBG PC and FC, a decrease in fluorescence intensity of the Calcofluor White channel compared to their respective HT control samples confirmed a reduction in visible OBG aggregates upon hydrolysis (Supporting Information, Figure S10). Notably, reduced light scattering and UV signals were observed in peak 2 after full cleavage of OBG (Figure 5). The reduced protein dispersibility in FC was corroborated by an increase in the number of visible protein aggregates observed in CLSM and a greater sample turbidity (Figure 5e). Integration of the OPBG FC Trp FLD fractograms also revealed a 15% and 64% reduction in signal relative to OPBG and OPBG PC respectively (Supporting Information, Figure S9).

Altogether, the AF4 analysis of enzyme treated OPBG revealed that increased dispersibility of oat globulin aggregates occurs after partial OBG cleavage, which acts synergistically to heat treatment. Conversely, the aggregation of protein in FC confirms that complete removal of OBG has a direct consequence on the protein dispersion. We later discuss the hypothesis that aggregation in FC stems from the promotion of protein–protein association via hydrophobic interactions upon removal of OBG.

### 3.3 Bulk and Surface Properties of Enzymatically Cleaved OPBG

To gain an understanding of the structure–function relationships governing OPBG, the bulk and surface properties after enzyme treatment were tested. OPI extracted from the same starting material (Figure 1aiii) was used as a reference since it is the most studied form of oat protein in the literature, thereby validating any potential benefits of using OPBG. For more information on the OPI used in this study, including the protein fractions present, the particle size distribution and the particle morphology, see Figure S11 in the Supporting Information.

The apparent viscosity of the samples was first measured to understand the aggregation state and structural rearragements of the oat protein dispersions under shear (Figure 6a). All OPBG samples exhibited Newtonian behavior and low viscosity across the shear rates tested due to the dilute nature of the samples (0.5% w/v). Although HT samples showed identical viscosity to OPBG (2 mPa.s), enzyme treatment resulted in a slight decrease in viscosity in FC and PC samples (1 mPa.s). OPI displayed similar viscosity to OPBG at high shear rates (1 mPa.s, 100 s^–1^), however it exhibited an apparent shear thinning behavior which was not observed with other samples. Given that the protein concentration was kept the same in all samples tested, we suggest that this difference stems either from the break up of large OPI aggregates during the experiment or is an artifact derived from the interfacial contributions^50^ of the isolated protein.

**Figure 6:**
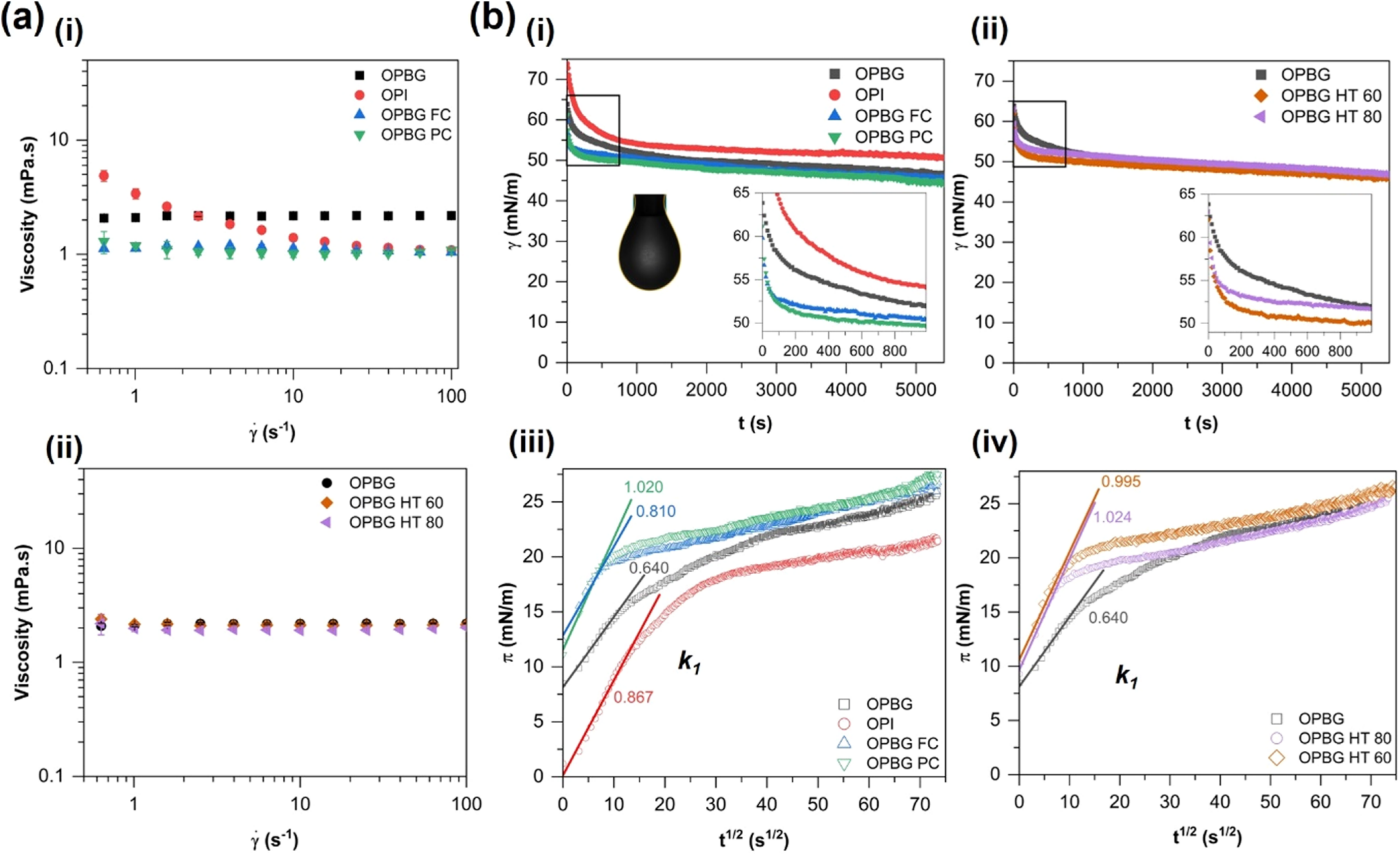
Apparent viscosity of oat protein samples and their surface behavior at the air–water interface. All samples were measured at an equal concentration of protein (4.4 mg/mL). (a) Flow curves of oat protein samples were measured at 25 °C and plots are shown as the mean of three measurements (*n* = 3) from independently prepared samples. (b) Air–water interfacial tension (γ) measurements as a function of time (*t*) are plotted in (i) and (ii). Data shows the mean of three measurements (*n* = 3), with at least one data set derived from an independently prepared sample. Inset is an example image captured of a 20 μL OPBG droplet being monitored a*t t* = 0 s. Plots (iii) and (iv) show the fitting of the first stage of protein adsorption at the air–water interface, i.e., the diffusion stage (*k*_1_).

We next investigated how hydrolysis of OBG affects the surface behavior at the air–water interface (Figure 6b). Notably, all OPBG samples exhibited a lower surface tension (44–45 mN/m) compared to OPI (51 mN/m) at the same protein concentration. An apparent change in the kinetics of absorption resulted in the fitting of the interfacial data into three stages of adsorption.^36^ The diffusion stage (*k*_1_) depicts the diffusion limited movement of protein from the bulk phase toward the interface. This is followed by penetration of protein at the interface (*k*_2_) and subsequent rearrangement of protein at the adsorbed layer (*k*_3_). Although *k*_2_ and *k*_3_ showed only subtle differences between the samples tested (Supporting Information, Figure S12), we found in Figure 6b that the value of *k*_1_ increased in the order: OPBG < OPBG FC < OPI < OPBG PC ∼ OPBG HT.

To understand these differences in surface adsorption kinetics, dispersion stability photographs of the samples were taken over a 24 h period (Supporting Information, Figure S13). The sedimentation over 24 h (%) was calculated from the dispersion stability and plotted against the integrated Trp FL area obtained from the AF4 fractograms, resulting in a linear correlation of R^2^ > 0.9 (Supporting Information, Figure S9). Results confirmed the increased dispersibility of OPBG samples in the order: OPBG FC < OPBG < OPBG PC.

The relative surface hydrophobicity index (H_0_) using fluorescent probe ANS (Supporting Information, Figure S14) was also measured. Despite the slight increase in hydrophobicity of HT samples compared to control, the relative surface hydrophobicity index (H_0_) did not increase after full cleavage. We propose that this is a result of ANS binding exclusively to the exposed hydrophobic surface area, rather than measuring the total hydrophobic content of the sample. Previous work established that OBG does not bind tightly to the hydrophobic proteinaceous regions in OPBG, but only loosely associates with the protein.^12^ Since ANS is a relatively small molecule with a high diffusion coefficient, the protein remains equally accessible to the dye before and after hydrolysis of OBG, resulting in no change to the measured H_0_ using this method.

The most striking difference in dispersion stability and surface behavior arises when comparing OPBG samples to OPI. The latter showed significantly reduced dispersion stability (Supporting Information, Figure S13) with a calculated sedimentation of 14.8% over 24 h, resulting in a 6-fold increase compared to OPBG (2.5%). This is supported by a 6-fold increase in the measured H_0_ for OPI compared to OPBG (Supporting Information, Figure S14). Since freeze-drying OPBG did not considerably affect OPBG dispersion stability relative to OPI (Supporting Information, Figure S13), we attribute the observed differences to the absence of the IEP step in the preparation of OPBG (Figure 1a). The presence of hydrophilic OBG and its fragments in OPBG samples may act as a steric barrier to limit hydrophobic protein–protein interactions, thus increasing the resulting dispersion stability.

The highly aggregated state of OPI explains the higher surface tension obtained in Figure 6bi despite its increased protein purity (95%). We propose that extreme precipitation of protein during the time frame of the run likely limits further lowering of the surface tension. The two linear regions observed before the system reached steady state in Figure 6iii may also indicate the presence of a faster and a slower diffusion phase, i.e. an initial *k*_*1*_ value dominated by smaller aggreagates, and a second, slower phase involving the diffusion of highly aggregated OPI species.

Figure 7 shows the adsorption of OPBG samples onto a polydimethylsiloxane (PDMS)-coated hydrophobic surface. The raw data in (a–f) was used to report the final values of −Δ*f* (Figure 7f), which is representative of the total mass adsorbed onto the PDMS surface after the final buffer rinse. The time taken to reach equilibrium (Figure 7g) was also plotted as an indication of the adsorption kinetics. OPBG (Figure 7a) showed a strong adsorption (−Δ*f* = 27 Hz) profile onto PDMS with low values of dissipation (Δ*D* = 1.3 ppm), the latter indicating the formation of a thin rigid film at the PDMS surface. In contrast, OPI displayed a stronger (−Δ*f* = 38 Hz) but significantly slower (*p* < 0.001) rate of adsorption, consistent with extremely aggregated protein, and did not reach a state of equilibrium after being left to adsorb for over 3.5 h (Figure S15, Supporting Information).

**Figure 7:**
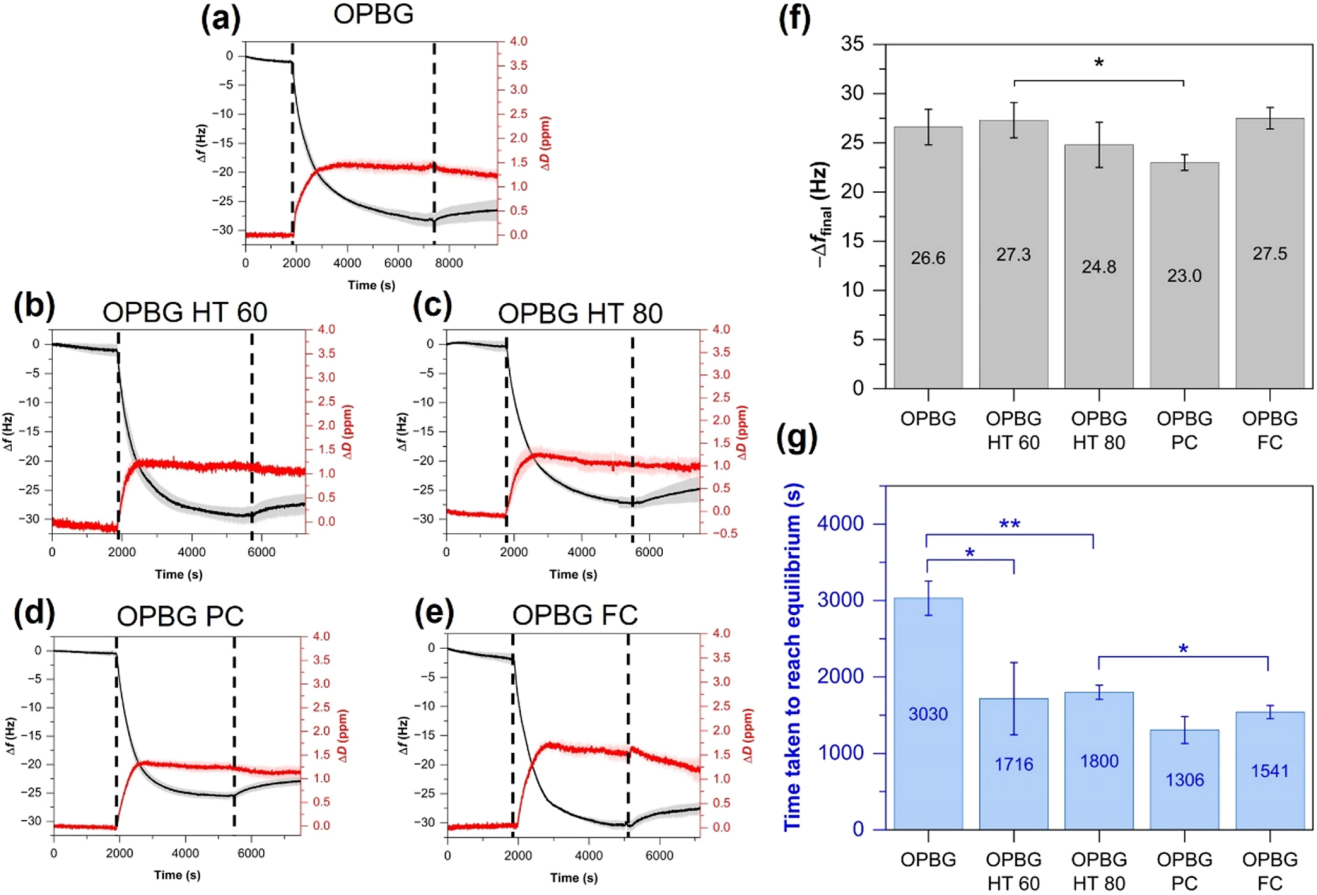
Adsorption of OPBG samples onto hydrophobic PDMS coated SiO_2_ surfaces using quartz crystal microbalance with dissipation monitoring (QCM-D). All samples were dissolved to a concentration of 0.01% w/v protein in HEPES buffer. Mean frequency (Δ*f*) and dissipation (Δ*D*) vs time plots for the 5th overtone showing the adsorption of (a) OPBG, (b) OPBG HT 60, (c) OPBG HT 80, (d) OPBG PC and (e) OPBG FC. After a stable baseline with HEPES buffer was obtained over 30 min, samples were introduced until an equilibrium state was achieved (change in Δ*f* < 2 Hz for 30 min). Shaded regions are the standard deviations obtained from three mesaurements (*n* = 3) obtained from two independent samples. A final HEPES buffer rinse was introduced to remove loosely attached particles. Bar chart comparison of (f) the time taken to reach an apparent equilibrium, i.e. Two Hz from the plateau frequency value prior to the final HEPES buffer rinse. The absolute mean frequency values after the buffer rinse are plotted in (g). Significant differences of *p* < 0.05 are marked with one asterisk (*), whereas *p* < 0.01 and *p* < 0.001 are marked with two (**) and three (***) asterisks, respectively.

A measurement of commercial OBG with a purity of >94%was conducted to confirm whether there was an effect of the isolated polysaccharide on the adsorption profiles shown (Supporting Information, Figure S15). As anticipated, commercial OBG showed limited adsorption (−Δ*f* = 5 Hz) due to its hydrophilic nature and limited association to the hydrophobic PDMS surfaces. These findings confirm that the adsorption profile of OPBG is driven by the protein and associated protein/OBG aggregates rather than isolated OBG. We observed little change in −Δ*f* after heat treatment of OPBG (Figure 7f), but significantly faster adsorption (Figure 7g) of HT samples compared to unheated OPBG. We attribute this to a loosening of the structure upon heat treatment, which leads to increased protein dispersibility (Supporting Information, Figure S9) and effectively increases the sample adsorption kinetics to the surface of the QCM-D sensors. Although the final −Δ*f* values for all samples tested remained mostly similar (Figure 7f), OPBG PC exhibited a significant decrease (*p* < 0.05) compared to HT 60. We hypothesize that this results from the break up of large OBG aggregates, which leads to a decrease in the water holding capacity of OBG.^51^ Since QCM-D measures the total effective hydrated mass, a decrease in −Δ*f* is observed when fewer water molecules become associated with OBG after PC.

In FC, −Δ*f* retains a similar value to HT 80 (27.5 Hz) despite reducing the hydrated mass associated with the fiber’s water retention. The time taken to reach an apparent equilibrium (Figure 7g) was also shorter in FC than in HT 80, indicating a faster adsorption in spite of FC being in a more aggregated state (Figure 5). We propose that upon full cleavage of OBG, a barrier to protein–protein aggregation is removed which results in the protein becoming effectively more hydrophobic. The increased effective hydrophobicity enhances the sample’s affinity for the hydrophobic PDMS surface, providing an explanation for the results in Figure 7 and the reduced dispersion stability of FC compared to OPBG and PC samples (Supporting Information, Figure S9).

In summary, the presence of minor proportions of OBG in oat protein achieved via partial enzymatic cleavage is a novel strategy to increase the dispersibility and enhance the surface behavior of oat protein. As illustrated in Figure 8, partial OBG hydrolysis releases proteins previously associated with aggregated OBG in OPBG, thereby increasing the availability of oat protein at the surface. Moreover, remnants of hydrophilic OBG in OPBG PC resulted in improved protein dispersibility with faster adsorption kinetics at the air–water and hydrophobic solid surface-water interfaces, where contribution of the thermal treatment required for enzyme hydrolysis cannot be ignored. In contrast, when the OBG fiber is fully hydrolyzed into glucose in FC, the steric hydrophilic barrier provided by OBG fragments is lost, thereby promoting oat protein aggregation and reducing protein dispersibility.

**Figure 8:**
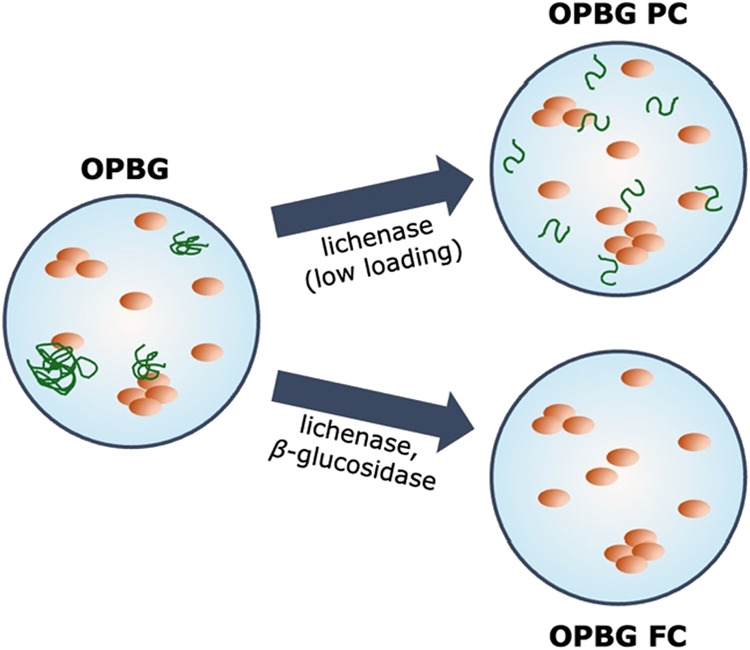
Schematic of OBG hydrolysis in OPBG leading to an improvement in dispersibility and surface behavior following partial cleavage (PC), while full cleavage (FC) leads to protein–protein hydrophobic aggregation. Oat protein is depicted as orange ellipses and OBG is represented as green flexible chains.

## 4 Limitations

Although the AF4 results in this study facilitate meaningful comparisons between the samples, they should be interpreted with caution. Retention times between UV/MALS and FLD set ups were not identical due to necessary differences in flow rates. A centrifugation step prior to analysis was necessary to prevent system blockages and the cross-flow was selected to detect all soluble protein. Therefore, information on larger quantities of the sample which consists of larger scale aggregates was lost in the analysis. Despite using two d*n*/d*c* values to minimize the issue of fitting an uncharacterized sample with multiple components coeluting, we recommend using the molar mass values cited in this study as indicative rather than absolute.

## 5 Conclusions

In this study, an enzymatic approach was used to investigate whether OBG influenced the protein dispersion in a previously characterized oat protein-rich extract (OPBG). An increase in protein dispersibility and solubilization were observed upon partial cleavage of OBG, with the heat-treatment stage being a prominent, synergistic driving force behind the changes observed. Notably, full cleavage of OBG resulted in protein aggregation, exhibiting poorer dispersion stability and slower kinetics of adsorption at the air–water interface compared to the heat-treated control. Based on these findings, we confirm that the presence of OBG in OPBG positively influences the oat protein dispersion and can act as a barrier to protein–protein aggregation under the specific sample composition, processing and conditions tested (neutral pH, low ionic strength).

All samples were also compared to OPI, which differs to OPBG by an additional isoelectric precipitation and freeze-drying step during extraction. Our findings confirm previous reports that OPI exhibits poor surface behavior. We attribute this to extreme protein–protein aggregation, which is driven by hydrophobic interactions. Remarkably, all OPBG samples tested showed a considerable increase in protein dispersion stability and functionality at surfaces, i.e. further lowering of surface tension at the air–water interface, and faster absorption kinetics onto hydrophobic surfaces compared to OPI. Since freeze-drying OPBG did not affect the improvements in dispersion stability (Supporting Information, Figure S13), our hypothesis is that these properties result from a combination of removing the isoelectric precipitation step, which likely influences the protein aggregation state upon rehydration, and retaining hydrophilic OBG in the system, which acts as a barrier to hydrophobic protein–protein aggregation. Further work is needed to evaluate the performance of OPBG in more complex and realistic systems, such as emulsions, which represent many food applications.

## Supplementary Material



## Notes

Additional experimental materials and methods; additional AF4 fractograms, including molar mass analysis and data corrections; circular dichroism spectroscopy data and fitting; SDS-PAGE gel images of OPBG after heat and enzyme treatment; fluorescence intensity measurements from confocal microscopy images; characterization of OPI compared to OPBG, including SDS-PAGE, particle size and confocal microscopy images; additional adsorption fitting of air–water interfacial tension data; relative surface hydrophobicity measurements; dispersion stability photographs of samples over 24 h; and QCM-D adsorption data of OPI and commercial OBG (PDF)
