# Supplementary material for: Unravelling the Role of Oat β‑Glucan on the Surface Behavior of an Oat Protein-Rich Coextract: An Enzymatic Approach
Source: Biomacromolecules. 2026 Jun 11;27(7):4382–94. doi: 10.1021/acs.biomac.6c00335 (PMC13370778; doi:10.1021/acs.biomac.6c00335)
Supplement: Supplementary file 1 [file bm6c00335_si_001.pdf]

Supporting Information

Unravelling the role of oat beta-glucan on the surface behaviour of an oat protein-rich co-extract: an enzymatic approach

*Jennifer McLauchlan[1***], Hans Bolinsson[2], Lars Nilsson[2**], Nick Sirijovski[3], Arwen I.I. Tyler[1], Caroline Orfila[3], and Anwesha Sarkar[1,4*]*

[1] Food Colloids and Bioprocessing Group, School of Food Science and Nutrition, University of Leeds, Leeds, LS2 9JT, United Kingdom.

[2] Department of Process and Life Science Engineering, Faculty of Engineering LTH, Lund University, Lund, 223 62, Sweden.

[3] Global Oatly Science and Innovation Centre, Oatly AB, Science Village, Lund, 221 00, Sweden.

[4] National Alternative Protein Innovation Centre (NAPIC), Leeds, LS2 9JT, United Kingdom.

Corresponding authors:

*A.Sarkar@leeds.ac.uk (A. Sarkar).

** lars.nilsson@ple.lth.se (L. Nilsson)

*** mmjam@leeds.ac.uk (J. McLauchlan)

Number of pages: 18

Number of figures: 14

Number of tables: 1

Contents

Methods for Supplementary data

Protein concentration

Circular dichroism spectroscopy

Dynamic light scattering

Sodium dodecyl sulphate-polyacrylamide gel electrophoresis (SDS-PAGE)

Relative surface hydrophobicity index

Supplementary data

Figure S1. AF4 fractograms using the in-line Calcofluor White fluorescence detection (FLD) set up to tune the enzyme reaction conditions for partial cleavage of OPBG. In (a), two concentrations of lichenase were tested (0.001 U/mL and 0.1 U/mL) with a constant incubation time of 60 min at 40° C. In (b), the concentration was kept the same (0.001 U/mL) and the effect of increasing the enzyme incubation time at 40 ˚C was monitored.

Figure S2. Populations obtained from the UV/MALS and FLD setups in Figures 3, 4 and 5 following transformation of the FLD retention times. Retention times were transformed AF4 theory[1] by linear interpolation from the relationship between analyte diffusion coefficient and retention time. Shaded areas represent data obtained post-elution after the cross flow was set to zero.

Figure S3. Effect of sample centrifugation prior to AF4 analysis on (a) protein concentration, measured using the Bradford assay method, and (b) the visual appearance of OPBG samples before and after centrifugation, in (i) and (ii) respectively. Bradford assay measurements were taken as three repeats in two independent experiments ($n = 3 \times 2$).

Figure S4. AF4 fractograms showing the intrinsic protein Trp fluorescence of (a) OP precursor and (b) OPBG samples captured using the FLD set up. Spectra for (a) and (b) were captured using different fluorescence gain

settings, since a lower gain was necessary in (a) to prevent signal cut off. The dataset was truncated at 60 min, as only this portion of the signal was used for analysis.

Figure S5. Raw data and fitting for circular dichroism (CD) spectroscopy secondary structure analysis. (a) Raw CD data for (i) heat-treated control samples and (ii) components of OPBG, *i.e.*, oat protein isolate (OPI) and oat *β*-glucan (>94%, Megazyme, Bray, Ireland). Each sample was measured in triplicate and plotted as an average ($n = 3 \times 1$). (b) Secondary structure fitting (red line) of the obtained CD data (black circles) using BestSel web server. Noise was observed in OPBG heat-treated for 60 and 80 min (OPBG HT 60 and HT 80, respectively) at wavelengths below 205 nm (arrows) resulting from HEPES buffer. Therefore, only the fitting for HT 120 was used to determine whether protein denaturation took place upon heating. (c) Secondary structure values obtained for OPBG and OPBG HT 120 (maximum heating time used) from the fitting in (b).

Figure S6. Sodium dodecyl sulphate polyacrylamide gel electrophoresis (SDS-PAGE) gels obtained under reducing (R) and non-reducing conditions (NR) of OPBG samples before and after heat treatment (HT). Samples after enzyme treatment are labelled as partially cleaved (PC) and fully cleaved (FC).

Figure S7. Radius of gyration (R_g) and molar mass (*MM*) analysis of multi-angle light scattering (MALS) plots for heat-treated controls and enzyme-treated OPBG samples. Values were fitted with the Berry method using two dn/dc values: bovine serum albumin (0.185 mL/g) and pure oat *β*-glucan (0.146 mL/g).

Table S8. Molar mass and root-mean-square radius values obtained from fitting of multi-angle light scattering (MALS) data at peak maxima using dn/dc values of (a) pure oat *β*-glucan (0.146 mL/g) and (b) bovine serum albumin (0.185 mL/g).

Figure S9. Dispersibility of OPBG following partial and complete hydrolysis of OBG. In (a), integrated Trp fluorescence (FL) signals for OPBG, PC, and FC were calculated after baseline subtraction and presented as a bar chart. Sedimentation after 24 h (red circles) was determined from dispersion stability images shown in Figure S15. Briefly, vial dimensions were used to calculate sediment height from the images using Fiji ImageJ software. This height was converted to sediment volume and expressed as a percentage (%) of the total sample volume in each vial. In (b), dispersibility values obtained from both methods are plotted, showing a strong linear correlation ($R^2 > 0.9$). The arrow indicates the direction of increasing protein dispersibility.

Figure S10. Calcofluor White fluorescence intensity measurements of heat-treated control samples and enzyme treated samples obtained from confocal laser scanning micrographs. Binary images of Calcofluor White channel were created using ImageJ of with a set threshold value of 75 to 255. Five images from each sample were analysed and shown in (a) OPBG HT 80 (b) OPBG HT 60 (c) OPBG FC and (d) OPBG PC. Determination of mean grey value and integrated density in (e) and (f) were conducted using an average of the five images from each sample.

Figure S11. Protein fractions, size distribution and morphology of oat protein isolate (OPI) compared to OPBG. (a) Sodium dodecyl sulphate polyacrylamide gel electrophoresis (SDS-PAGE) gels obtained under reducing (R) and non-reducing conditions (NR) for OPI and OPBG. (b) Dynamic light scattering (DLS) size distribution of OPI and OPBG. Samples were prepared at 4.4 mg/mL protein then centrifuged under the same conditions as those used prior to AF4 injection (13,000 rpm, 30 mins, rt). The supernatant was collected and the resulting hydrodynamic diameter (d_H) distribution as a function of scattering intensity was plotted. An average of three measurement from one sample ($n = 3 \times 1$) was plotted. (c) Confocal laser scanning micrographs of OPI and OPBG. Channel (i) shows imaging under white light. Samples were stained with fast green (FG) and calcofluor white (CW) using excitation wavelengths of (i) 633 nm and (ii) 380 nm respectively. Fluorescence channels (ii) and (iii) were merged to create (iv).

Figure S12. Fitting of the second and third stages of oat protein adsorption at the air-water interface. Graph (a) and (b) shows the linear fit of the penetration stage (k_2) obtained using OriginPro. Plots (c) and (d) is the linear fit used to determine the rate of rearrangement at the interface (k_3). Both stages are also depicted schematically, with orange circles representing globular proteins and blue lines representing oat β-glucan.

Figure S13. Dispersion stability of OPBG and OPI samples over a period of 24 h. Photographs at 0 h were obtained directly after mixing and samples were stored at room temperature (~20 °C) throughout. In (a), OPBG was dialysed against HEPES buffer and frozen in liquid form as single use aliquots and defrosted prior to testing. OPI was prepared at 4.5 mg/mL protein in HEPES buffer and left stirring for 2 h prior to photographs. In (b), OPBG was dialysed against water and freeze dried into a powder to understand the effect of lyophilisation on the dispersion stability. Both OPI and OPBG were prepared at a protein concentration of 5 mg/mL protein in HEPES buffer and left stirring at 700 rpm for 2 h to solubilise prior to photographs.

Figure S14. Relative surface hydrophobicity (H_0) measurements obtained with fluorescence probe 8-anilo-1-napthalenesulphonic acid (ANS). A serial dilution containing a minimum of 5 concentrations for each sample was prepared and three samples of each concentration were measured in triplicate ($n = 3 \times 3$). In (a), plots of fluorescence intensity vs. protein concentration are shown. The slope of the linear fits shown in (a) were used to obtain the H_0 values plotted in (b).

Figure S15. Adsorption of (a) OPI (0.01% w/v protein) and (b) OBG (0.01% w/v) onto hydrophobic PDMS coated SiO_2 surfaces using quartz crystal microbalance with dissipation monitoring (QCM-D). Figures (a) and (b) show frequency (Δf) and dissipation (ΔD) vs. time plots for the 5th overtone. After a stable baseline with HEPES buffer was obtained over 30 min, samples were introduced until an equilibrium state was achieved (change in $\Delta f < 2$ Hz for 30 min). A final HEPES buffer rinse was introduced to remove loosely attached particles. Shaded regions are the standard deviations from three separate sensors obtained in one independent experiment (n = 3 x 1). The absolute mean frequency values obtained after the final ($-\Delta f_{final}$) buffer rinse are compared to OPBG in (c).

References

Methods for Supplementary data

Protein concentration

The reported protein concentration in Figure S1 was measured using the Pierce™ Bradford Protein Assay Kit (Thermo Fisher Scientific, Waltham, MA, USA). All absorbance measurements were read at 595 nm using a Multiskan™ FC microplate photometer and SkanIt™ software version 7.0.2 (Thermo Fisher Scientific, Waltham, MA, USA). A standard curve with bovine serum albumin (BSA) was plotted (0.1-1.0 mg/mL) after a blank reading was subtracted from all measurements. OPBG was diluted with HEPES buffer to give absorbances within the calibration curve range. Measurements for all standards and samples were conducted in triplicate and incubated at room temperature for approximately 10 min prior to absorbance measurements.

Circular dichroism (CD) spectroscopy

Far-UV CD measurements (180 to 260 nm) were acquired in triplicate using a Chirascan Plus CD spectrometer (Applied Photophysics, Surrey, UK) in steps of 1 nm with a bandwidth of 2.0 nm at 20 °C. Samples and 10 mM HEPES (pH 7.0) buffer were pipetted into a cuvette with a path length of 1.0 mm. Spectra were plotted as the difference between the average sample signal and the average buffer signal. Secondary structure analysis and fitting in the wavelength region of 190 to 250 nm was conducted using the BeStSel web server[2, 3] (http://bestsel.elte.hu). Regions below 190 nm were not used for fitting due to noise arising from HEPES buffer.

Dynamic light scattering

Dynamic light scattering (DLS) with a backscattering angle of 173° was conducted using a ZetaSizer Ultra (Malvern Instruments Ltd., Worcestershire, UK). All solutions at 4.4 mg/mL protein were centrifuged using the asymmetric field-flow fractionation (AF4) preparation conditions (13,000 rpm, 30 min, rt) prior to insertion into a DTS1070 capillary cell (Malvern Instruments Ltd., Worcestershire, UK).

The hydrodynamic diameter (d_H) was calculated via the Stokes-Einstein equation (1), where D is the diffusion coefficient, T is the temperature and k_B is Boltzmann's constant (1). A refractive index value of 1.45, commonly used for protein extracts in solution was selected[4, 5] with an absorption of 0.001. The solvent was set to water with an RI of 1.33 and a viscosity (η) of 0.8872 mPa.s at 25 °C. All d_H measurements were conducted at 25 °C after the samples

equilibrated for 120 s inside the instrument. All samples were measured in triplicate and an average intensity distribution graph was plotted using a general non-negatively constrained least squares (NNLS) algorithm generated by the ZS Xplorer software (Malvern Instruments Ltd., Worcestershire, UK).

$$d_H = \frac{k_B T}{3\pi\eta D} \tag{1}$$

Sodium dodecyl sulphate-polyacrylamide gel electrophoresis (SDS-PAGE)

Oat protein samples were diluted to 1 mg/mL protein in HEPES buffer and tested under reducing (R) and non-reducing (NR) conditions. Mixtures of sample with lithium dodecyl sulphate (LDS) sample buffer and either buffer or 0.5 M 1,4-dithiothreitol (DTT) were heated in a water bath at 70 °C for 10 mins. After cooling, samples were loaded onto precast gels in an Invitrogen™ MiniGel Tank submerged in MES running buffer. Protein standard molecular weight markers were loaded onto the first and last lane. After running the gel at a constant voltage (200 V) for 30 mins, the gel was washed with water on a shaking table (3 × 5 min, 44 osc/min). The gel was subsequently stained and left on the shaking table overnight. After destaining by continuous washing with water, imaging and molecular weight analysis of the gel was conducted with a ChemiDoc™ XRS+ imaging system and Image Lab Software (Bio-Rad Laboratories, Richmond, CA, USA).

Relative surface hydrophobicity index

The relative surface hydrophobicity index (H_0) of oat protein-beta-glucan extract (OPBG) and oat protein isolate (OPI) samples in Figure S10 was determined using fluorescence probe 1-anilo-8-napthalenesulphonate (ANS), which binds to the hydrophobic regions of the sample that are exposed in solution. A serial dilution in 10 mM HEPES buffer (pH 7.0) was used to obtain concentrations ranging from 0.001 to 0.05 mg/mL protein. Next, 15 µL of ANS dye (8 mM in 10 mM HEPES, pH 7.0) was added to 3 mL of sample. Vials were carefully inverted, then left to incubate in the dark at room temperature for 15 minutes prior to measurement. A FluoroMax spectrofluorometer (Horiba, Northampton, UK) was used at a fixed excitation wavelength of 370 nm to capture emission spectra between 440 and 650 nm. Maximum fluorescence intensity values were then plotted as a function of protein concentration and a linear fit was used to obtain the slope (H_0). Measurements were performed in triplicate for three individual samples prepared on the same day ($n = 3 \times 3$).

Supplementary data

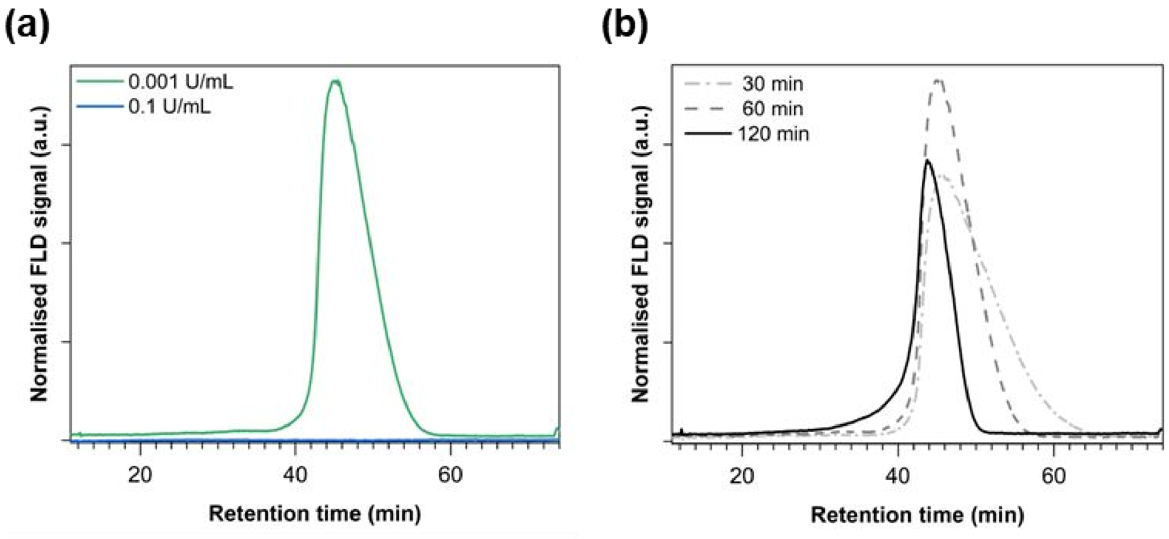

Figure S1. AF4 fractograms using the in-line Calcofluor White fluorescence detection (FLD) set up to tune the enzyme reaction conditions for partial cleavage of OPBG. In (a), two concentrations of lichenase were tested (0.001 U/mL and 0.1 U/mL) with a constant incubation time of 60 min at 40° C. In (b), the enzyme concentration was kept the same (0.001 U/mL) and the effect of increasing the enzyme incubation time at 40 ˚C was monitored.

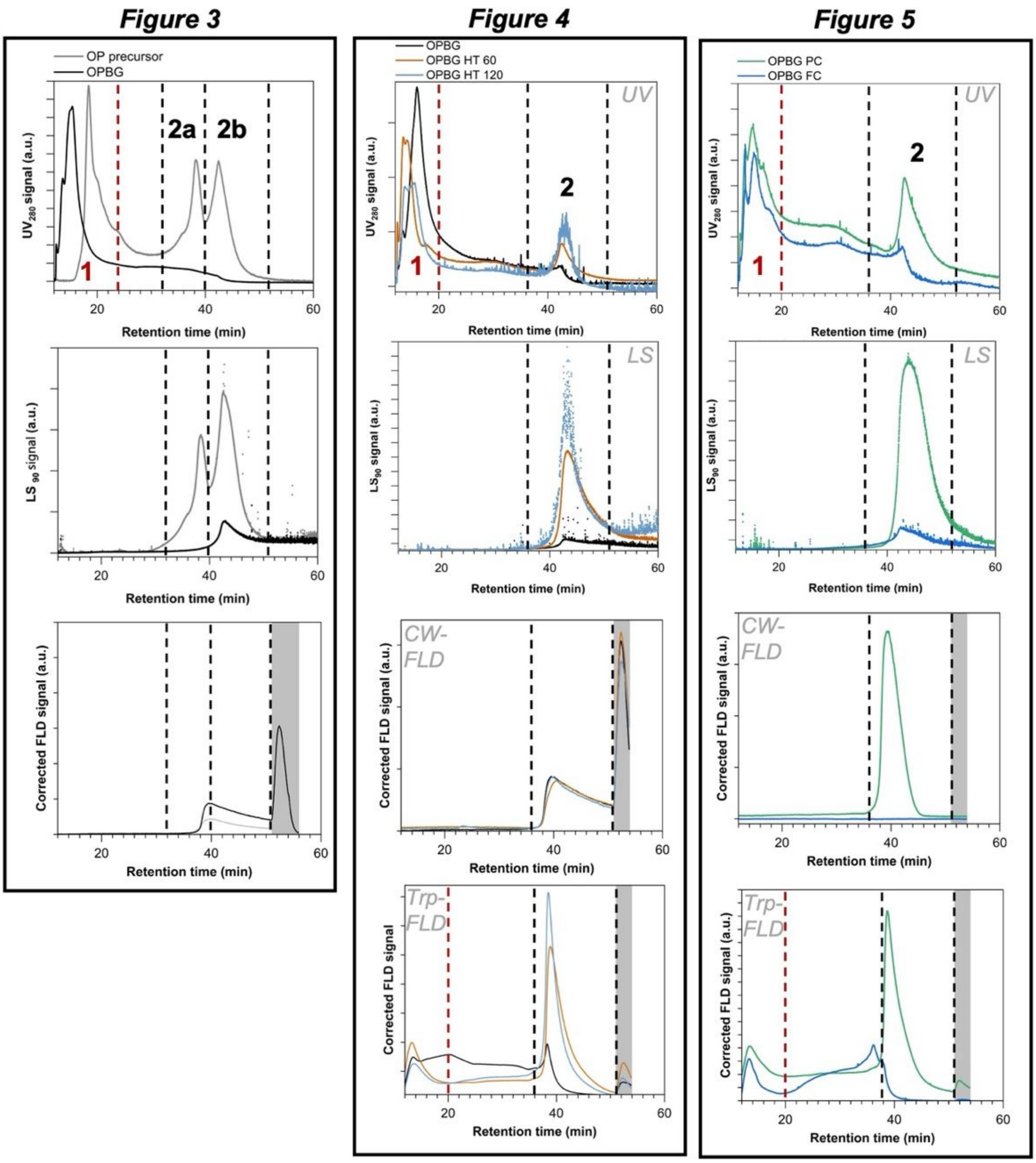

Figure S2. Populations obtained from the UV/MALS and FLD setups in Figures 3, 4 and 5 following transformation of the FLD retention times. Retention times were transformed AF4 theory[1] by linear interpolation from the relationship between analyte diffusion coefficient and retention time. Shaded areas represent data obtained post-elution after the cross flow was set to zero.

(a)

Sample	Measured protein concentration (mg/mL)
OPBG	4.4 ± 0.1
OPBG centrifuged	0.21 ± 0.04

(b)

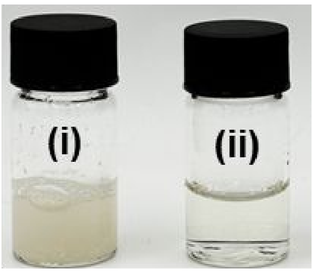

Figure S3. Effect of sample centrifugation prior to AF4 analysis. (a) Protein concentration before and after centrifugation, measured using the Bradford assay method. Bradford assay measurements were taken as three repeats in two independent samples ($n = 3 \times 2$). (b) Visual appearance of OPBG samples before and after centrifugation, in (i) and (ii) respectively.

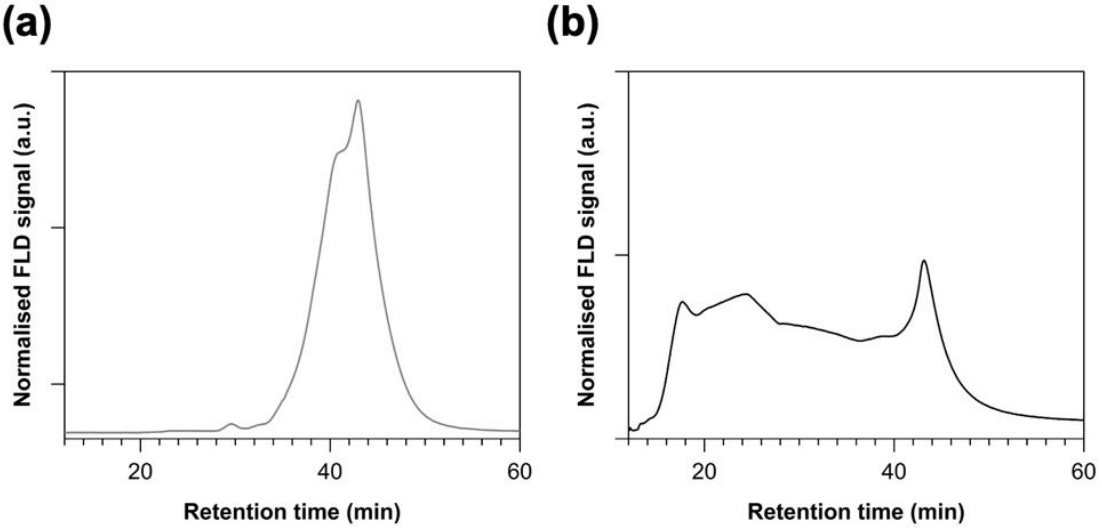

Figure S4. AF4 fractograms showing the intrinsic protein Trp fluorescence of (a) OP precursor and (b) OPBG samples captured using the FLD set up. Spectra for (a) and (b) were captured using different fluorescence gain settings, since a lower gain was necessary in (a) to prevent signal cut off. The dataset was truncated at 60 min, as only this portion of the signal was used for analysis.

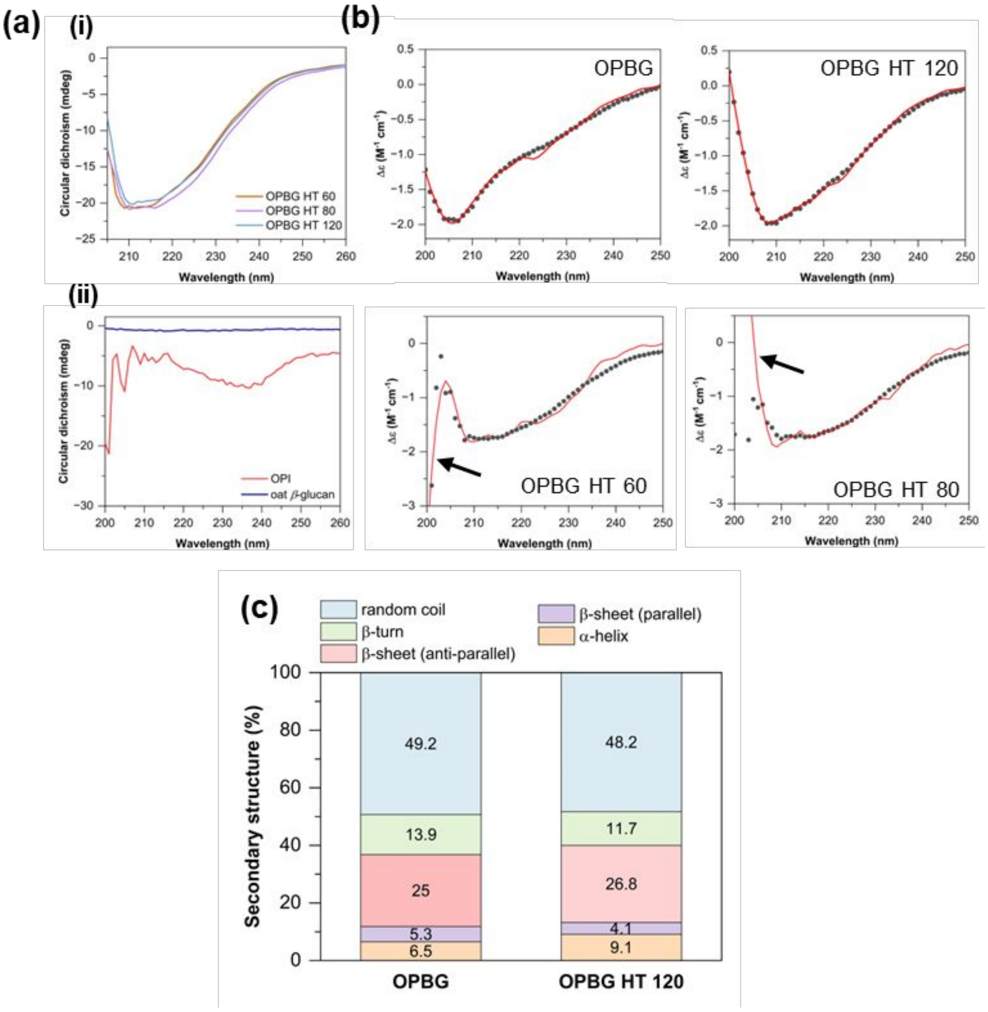

Figure S5. Raw data and fitting for circular dichroism (CD) spectroscopy secondary structure analysis. (a) Raw CD data for (i) HT control samples and (ii) components of OPBG, *i.e.*, oat protein isolate (OPI) and commercial OBG (>94%, Megazyme, Bray, Ireland). Each sample was measured in triplicate and plotted as an average (n = 3). (b) Secondary structure fitting (red line) of the obtained CD data (black circles) using BestSel web server. Noise was observed in OPBG HT 60 and 80 at wavelengths below 205 nm (arrows) resulting from HEPES buffer. Therefore, only the fitting for HT 120 was used to determine whether protein denaturation took place upon heating. (c) Secondary structure values obtained for OPBG and OPBG HT 120 (maximum heating time used) from the fitting in (b).

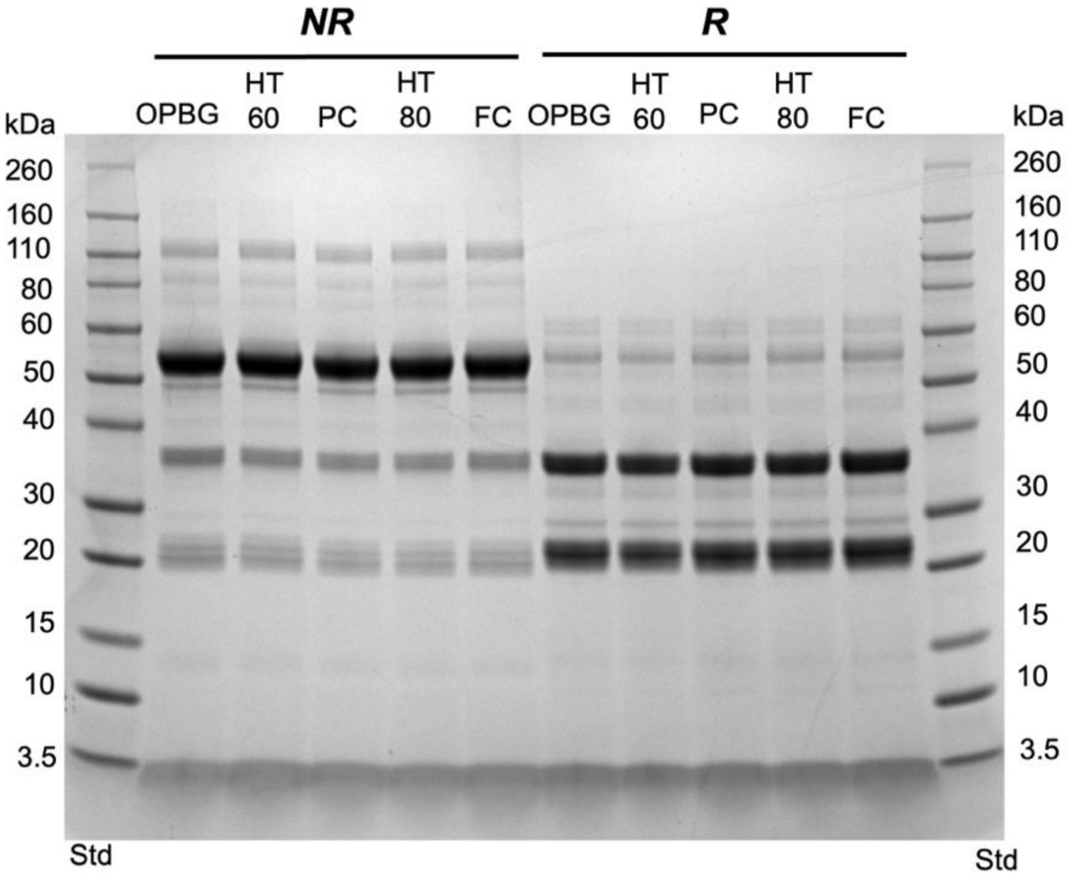

Figure S6. Sodium dodecyl sulphate polyacrylamide gel electrophoresis (SDS-PAGE) gels obtained under reducing (R) and non-reducing conditions (NR) of OPBG before and after heat treatment (HT). Samples after enzyme treatment are labelled as partially cleaved (PC) and fully cleaved (FC).

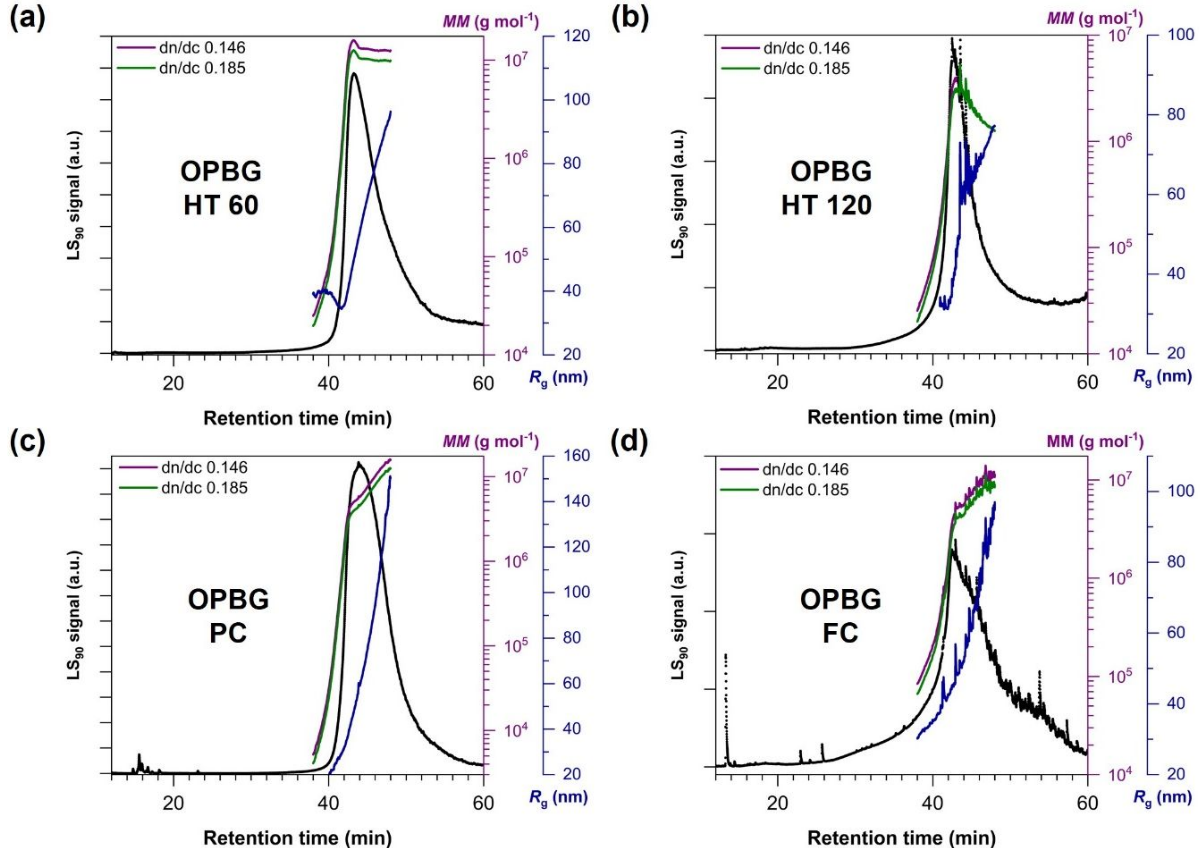

Figure S7. Radius of gyration (R_g) and molar mass (*MM*) analysis of multi-angle light scattering (MALS) plots for heat-treated controls and enzyme-treated OPBG samples. Values were fitted with the Berry method using two dn/dc values: bovine serum albumin (0.185 mL/g) and pure oat *β*-glucan (0.146 mL/g).

Table S8. Molar mass and root-mean-square radius values obtained from fitting of multi-angle light scattering (MALS) data at peak maxima using dn/dc values of (a) pure oat *β*-glucan (0.146 mL/g) and (b) bovine serum albumin (0.185 mL/g).

Sample	Peak maxima / min	Radius of peak at maxima / nm	Molar mass of peak at maxima / g mol^{-1}	
			(a) dn/dc 0.146	(b) dn/dc 0.185
OP precursor	38.2 (1)<br>42.5 (2)	14.3 (1)<br>22.2 (2)	295 (1)<br>770 (2)	190 (1)<br>607 (2)
OPBG	43.5	49.6	1,481	1,083
OPBG HT 60	43.2	49.8	15,952	12,589
OPBG HT 120	42.5	38.8	3,224	2,544
OPBG PC	43.9	60.1	7,233	4,614

OPBG FC	42.5	45.2	3,615	3,108

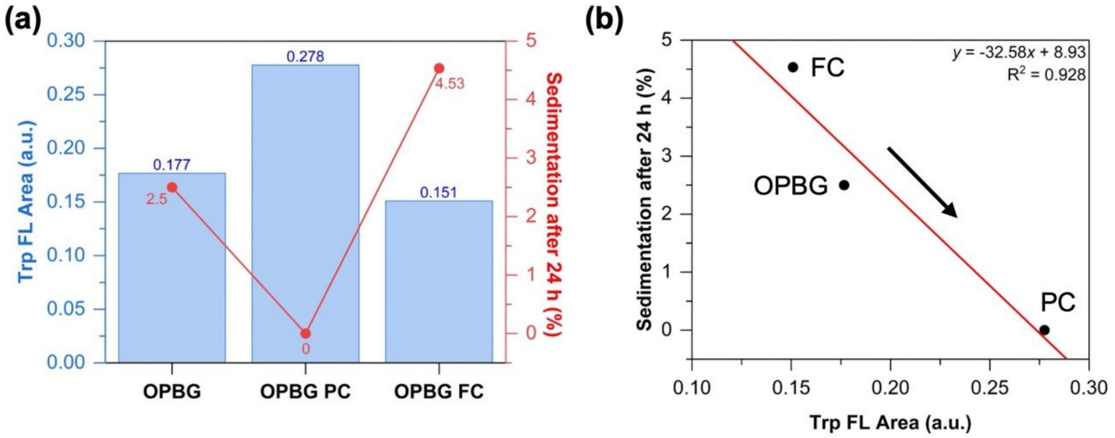

Figure S9. Dispersibility of OPBG following partial and complete hydrolysis of OBG. In (a), integrated Trp fluorescence (FL) signals for OPBG, PC, and FC were calculated after baseline subtraction and presented as a bar chart. Sedimentation after 24 h (red circles) was determined from dispersion stability images shown in Figure S15. Briefly, vial dimensions were used to calculate sediment height from the images using Fiji ImageJ software. This height was converted to sediment volume and expressed as a percentage (%) of the total sample volume in each vial. In (b), dispersibility values obtained from both methods are plotted, showing a strong linear correlation ($R^2 > 0.9$). The arrow indicates the direction of increasing protein dispersibility.

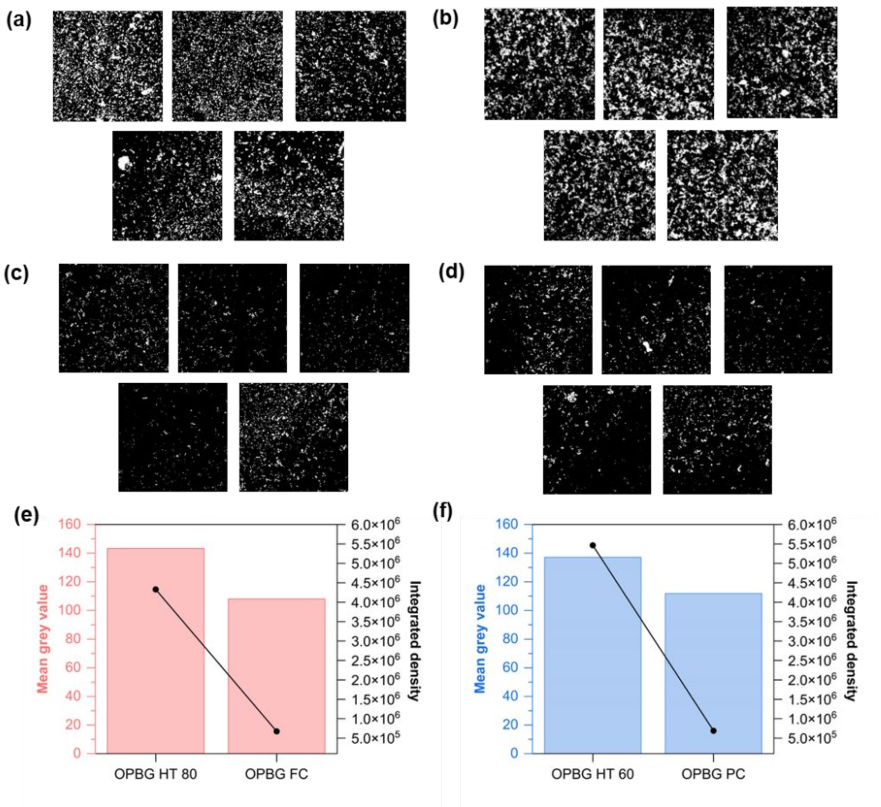

Figure S10. Calcofluor White fluorescence intensity measurements of heat-treated control samples and enzyme treated samples obtained from confocal laser scanning micrographs. Binary images of Calcofluor White channel were created using ImageJ of with a set threshold value of 75 to 255. Five images from each sample were analysed and shown in (a) OPBG HT 80 (b) OPBG HT 60 (c) OPBG FC and (d) OPBG PC. Determination of mean grey value and integrated density in (e) and (f) were conducted using an average of the five images from each sample.

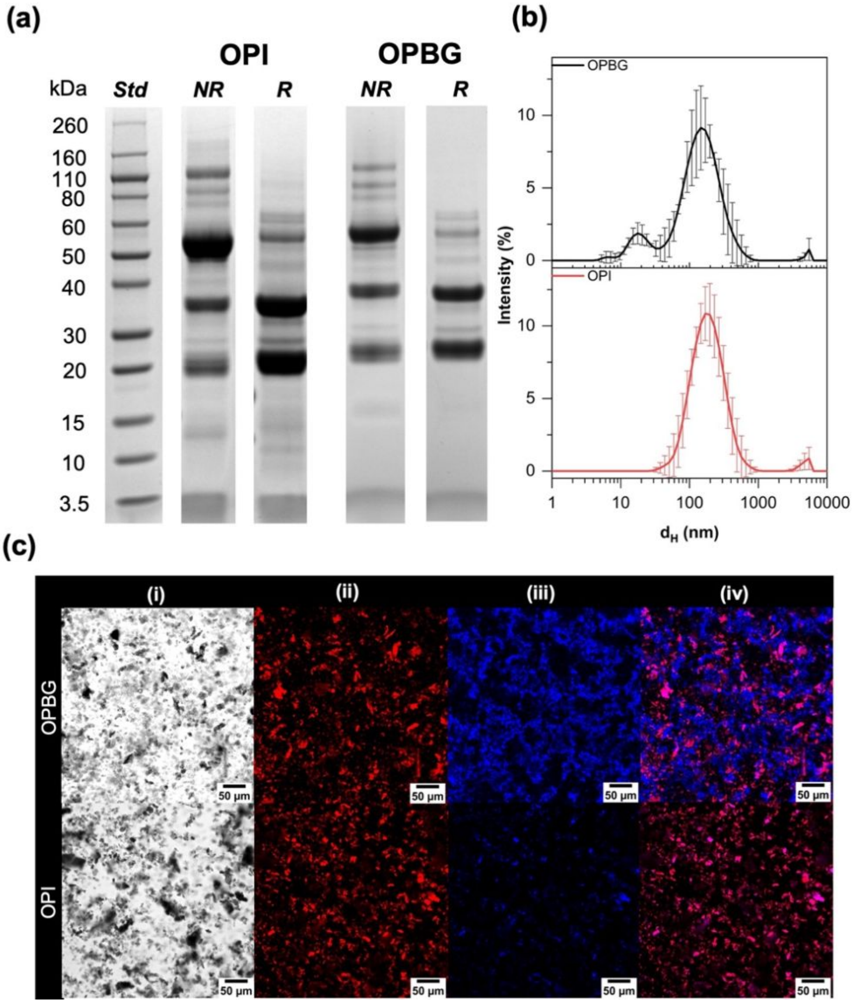

Figure S11. Protein fractions, size distribution and morphology of oat protein isolate (OPI) compared to OPBG. (a) Sodium dodecyl sulphate polyacrylamide gel electrophoresis (SDS-PAGE) gels obtained under reducing (R) and non-reducing conditions (NR) for OPI and OPBG. (b) Dynamic light scattering (DLS) size distribution of OPI and OPBG. Samples were prepared at 4.4 mg/mL protein then centrifuged under the same conditions as those used prior to AF4 injection (13,000 rpm, 30 mins, rt). The supernatant was collected and the resulting hydrodynamic diameter (d_H) distribution as a function of scattering intensity was plotted. An average of three measurement from one sample ($n = 3$) was plotted. (c) Confocal laser scanning micrographs of OPI and OPBG. Brightfield images are shown in (i). Samples were stained with fast green (FG) and calcofluor white (CW) using excitation wavelengths of (i) 633 nm and (ii) 380 nm respectively. Fluorescence channels (ii) and (iii) were merged to create (iv).

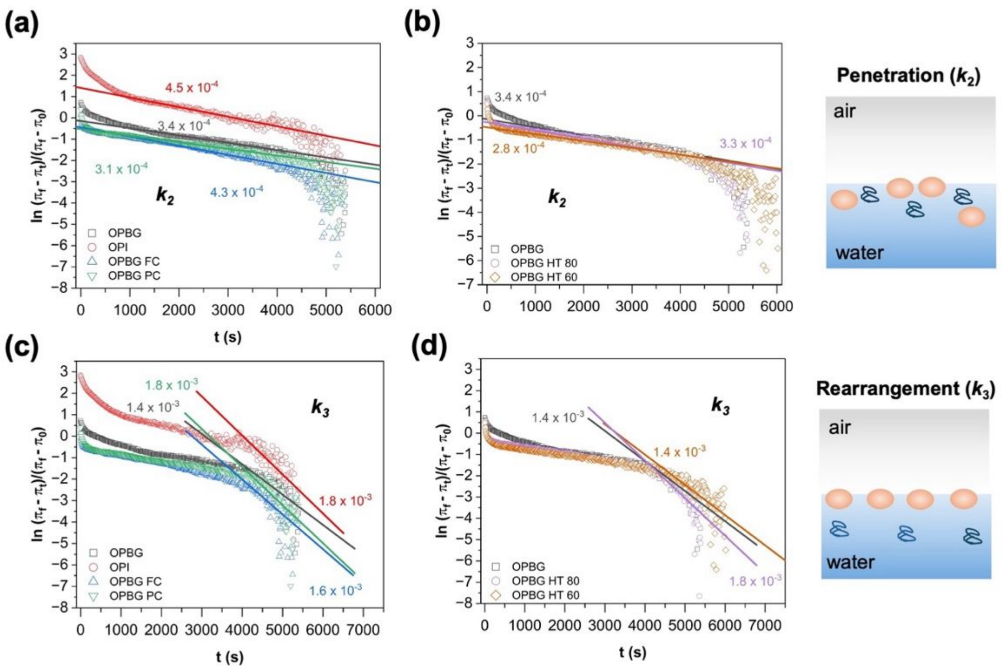

Figure S12. Fitting of the second and third stages of oat protein adsorption at the air-water interface. Graph (a) and (b) shows the linear fit of the penetration stage (k_2) obtained using OriginPro. Plots (c) and (d) is the linear fit used to determine the rate of rearrangement at the interface (k_3). Both stages are also depicted schematically, with orange circles representing globular proteins and blue lines representing OBG.

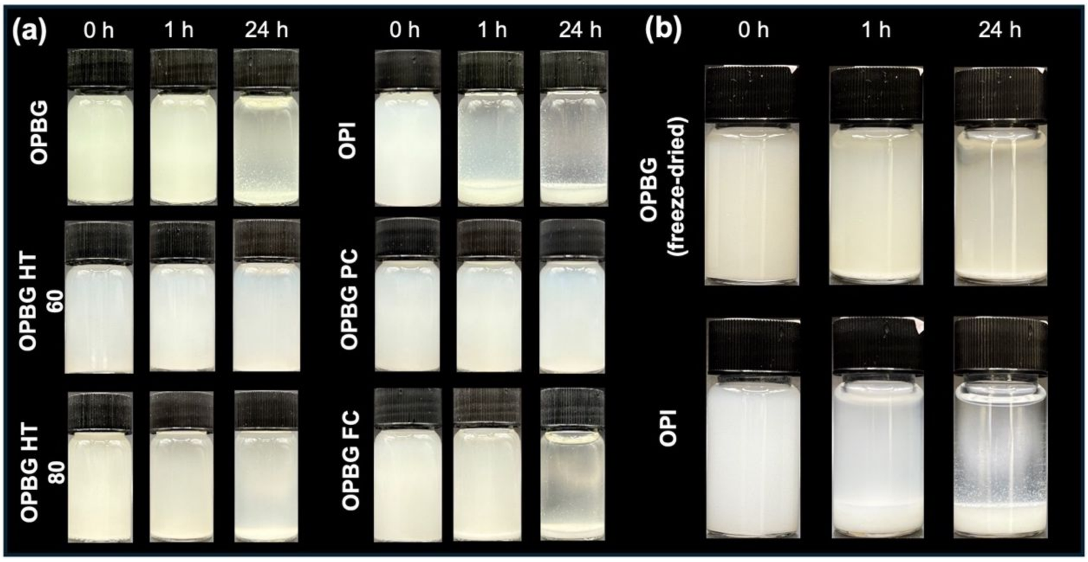

Figure S13. Dispersion stability of OPBG and OPI samples over a period of 24 h. Photographs at 0 h were obtained directly after mixing and samples were stored at room temperature (~20 ˚C) throughout. In (a), OPBG was dialysed against HEPES buffer and frozen in liquid form as single use aliquots and defrosted prior to testing. OPI was prepared at 4.5 mg/mL protein in HEPES buffer and left stirring for 2 h prior to photographs. In (b), OPBG was dialysed against water and freeze dried into a powder to understand the effect of lyophilisation on the

dispersion stability. Both OPI and OPBG were prepared at a protein concentration of 5 mg/mL protein in HEPES buffer and left stirring at 700 rpm for 2 h to solubilise prior to photographs.

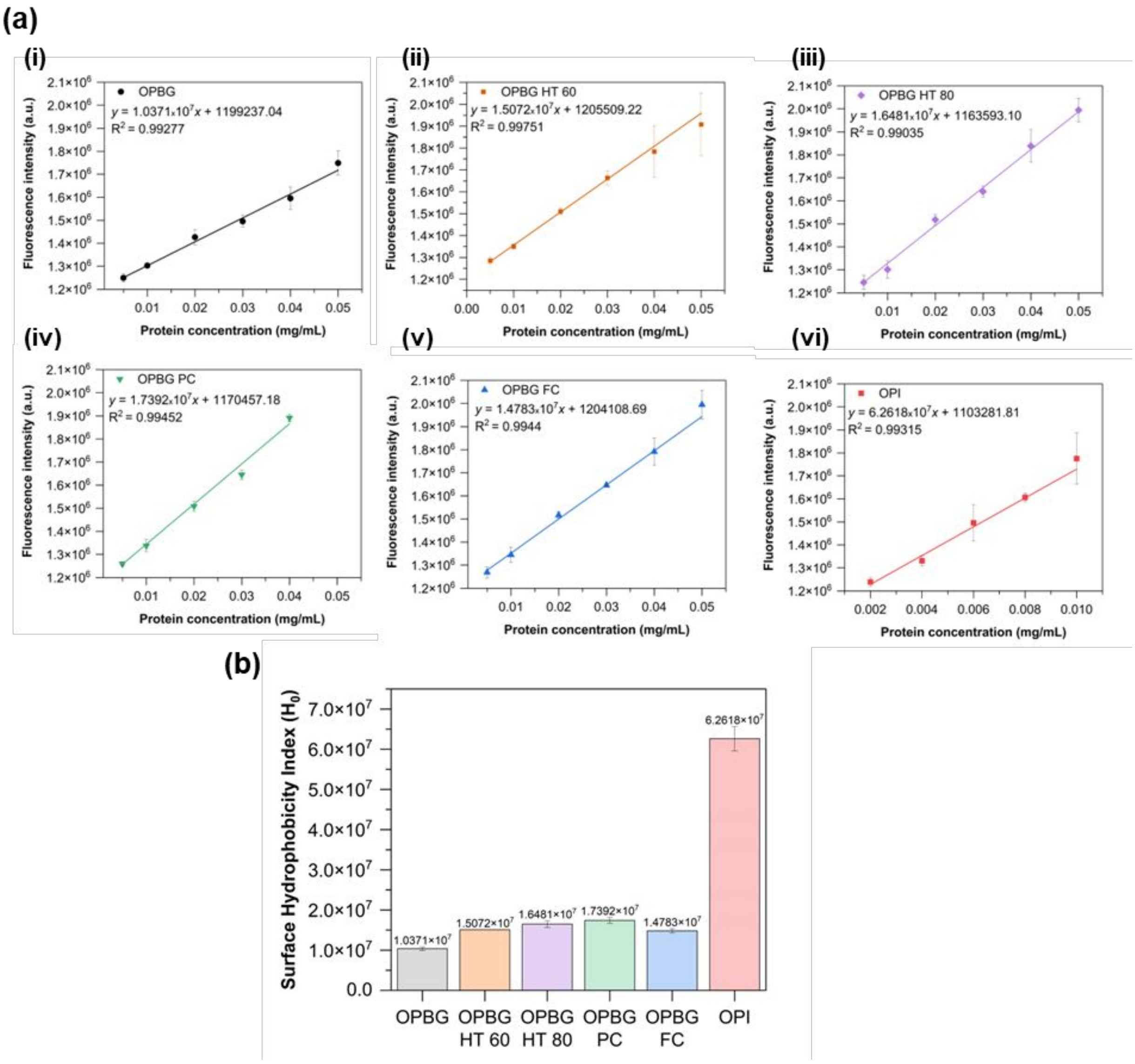

Figure S14. Relative surface hydrophobicity (H_0) measurements obtained with fluorescence probe 8-anilo-1-napthalenesulphonic acid (ANS). A serial dilution containing a minimum of 5 concentrations for each sample was prepared and three samples of each concentration were measured in triplicate ($n = 3 \times 3$). In (a), plots of fluorescence intensity vs. protein concentration are shown. The slope of the linear fits shown in (a) were used to obtain the H_0 values plotted in (b).

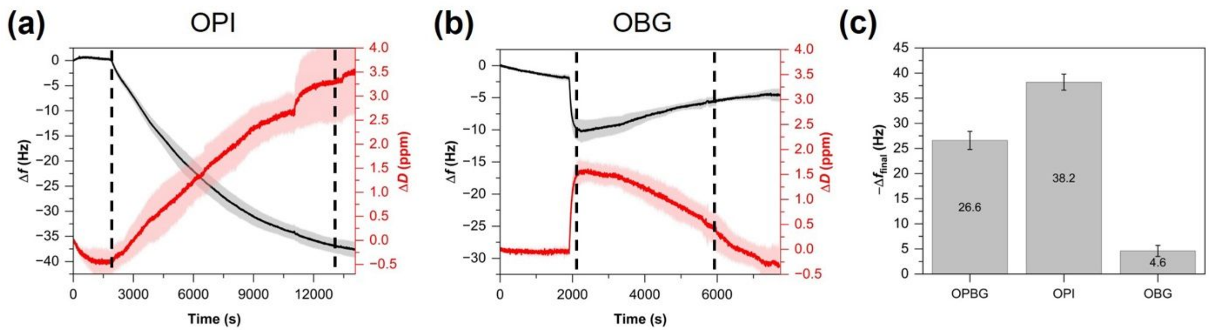

Figure S15. Adsorption of (a) OPI (0.01% w/v protein) and (b) OBG (0.01% w/v) onto hydrophobic PDMS coated SiO_2 surfaces using quartz crystal microbalance with dissipation monitoring (QCM-D). Figures (a) and (b) show frequency (Δf) and dissipation (ΔD) vs. time plots for the 5th overtone. After a stable baseline with HEPES buffer was obtained over 30 min, samples were introduced until an equilibrium state was achieved (change in $\Delta f < 2$ Hz for 30 min). A final HEPES buffer rinse was introduced to remove loosely attached particles. Shaded regions are the standard deviations from three separate sensors obtained in one independent experiment ($n = 3$). The absolute mean frequency values obtained after the final ($-\Delta f_{\text{final}}$) buffer rinse are compared to OPBG in (c).

References

(1) Håkansson, A.; Magnusson, E.; Bergenståhl, B.; Nilsson, L. Hydrodynamic radius determination with asymmetrical flow field-flow fractionation using decaying cross-flows. Part I. A theoretical approach. *J. Chromatogr. A* **2012**, *1253*, 120-126. DOI: 10.1016/j.chroma.2012.07.029.

(2) Micsonai, A.; Moussong, É.; Wien, F.; Boros, E.; Vadászi, H.; Murvai, N.; Lee, Y.-H.; Molnár, T.; Réfrégiers, M.; Goto, Y.; et al. BeStSel: webserver for secondary structure and fold prediction for protein CD spectroscopy. *Nucleic Acids Research* **2022**, *50* (W1), W90-W98. DOI: 10.1093/nar/gkac345.

(3) Micsonai, A.; Wien, F.; Bulyáki, É.; Kun, J.; Moussong, É.; Lee, Y.-H.; Goto, Y.; Réfrégiers, M.; Kardos, J. BeStSel: a web server for accurate protein secondary structure prediction and fold recognition from the circular dichroism spectra. *Nucleic Acids Research* **2018**, *46* (W1), W315-W322. DOI: 10.1093/nar/gky497.

(4) Amagliani, L.; Ben Sassi, E.; Buczkowski, J.; Schmitt, C. Influence of protein source on the morphology, physicochemical and flow properties of protein-based emulsion particles to be used as texture modulators. *Food Hydrocolloids* **2020**, *101*, 105581. DOI: 10.1016/j.foodhyd.2019.105581.

(5) de Vries, A.; Lopez Gomez, Y.; Jansen, B.; van der Linden, E.; Scholten, E. Controlling agglomeration of protein aggregates for structure formation in liquid oil: A sticky business. *ACS Appl. Mater. Interfaces* **2017**, *9* (11), 10136-10147. DOI: 10.1021/acsami.7b00443.
